# Stereochemical outcomes of C–F activation reactions of benzyl fluoride

**DOI:** 10.3762/bjoc.14.6

**Published:** 2018-01-09

**Authors:** Neil S Keddie, Pier Alexandre Champagne, Justine Desroches, Jean-François Paquin, David O'Hagan

**Affiliations:** 1 School of Chemistry, Biomedical Sciences Research Complex, University of St Andrews, North Haugh, St Andrews, Fife KY16 9ST, United Kingdom; 2 PROTEO, CCVC, Département de chimie, 1045 Avenue de la Médecine, Université Laval, Québec, QC G1V 0A6, Canada

**Keywords:** benzylic fluorides, C–F activation, chiral liquid crystal, ^2^H NMR, PBLG, stereochemistry

## Abstract

In recent years, the highly polar C–F bond has been utilised in activation chemistry despite its low reactivity to traditional nucleophiles, when compared to other C–X halogen bonds. Paquin’s group has reported extensive studies on the C–F activation of benzylic fluorides for nucleophilic substitutions and Friedel–Crafts reactions, using a range of hydrogen bond donors such as water, triols or hexafluoroisopropanol (HFIP) as the activators. This study examines the stereointegrity of the C–F activation reaction through the use of an enantiopure isotopomer of benzyl fluoride to identify whether the reaction conditions favour a dissociative (S_N_1) or associative (S_N_2) pathway. [^2^H]-Isotopomer ratios in the reactions were assayed using the Courtieu ^2^H NMR method in a chiral liquid crystal (poly-γ-benzyl-L-glutamate) matrix and demonstrated that both associative and dissociative pathways operate to varying degrees, according to the nature of the nucleophile and the hydrogen bond donor.

## Introduction

**Figure 1:**
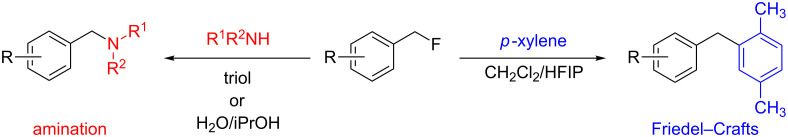
C–F activation of benzylic fluorides to generate benzylamine or diarylmethane products.

The C–F bond is the strongest carbon–halogen bond known [1]. Its low reactivity, in comparison to other C–X bonds, means that it is inert to all but the most harsh reaction conditions, and fluorine can generally be carried through multistep syntheses without concern over side reactions (the exception being S_N_Ar reactions). In recent years, there has been an increasing interest in C–F bond activation [2], with a view to using organic bound fluoride as a leaving group in substitution reactions that typically require more activated leaving groups. Such an approach could circumvent the requirement for protecting groups in multistep synthesis by capitalizing on the low reactivity of the C–F bond. Paquin et al. have published extensively on non-metal based methods for benzylic C–F bond activation [3–7]. The reactivity relies on protic activation driven by the capacity of organic fluoride to form hydrogen bonds [8–9]. Protocols using water/isopropanol [3], optimally coordinated triols [4–5], and hexafluoroisopropanol (HFIP) [6–7] as the corresponding hydrogen bond donors have shown considerable success. This mode of activation has been demonstrated for amination [3–5] and Friedel–Crafts reactions [6–7] on benzylic fluoride substrates (Figure 1), producing the corresponding substituted products in moderate to good yields. The water/isopropanol system was also shown to be amenable to phenolate and thiolate nucleophiles [3].

Previously, Paquin et al. undertook density functional theory (DFT) studies on the mechanism of C–F amination reactions employing water/isopropanol [3] and triols [4–5] as hydrogen-bond donor activators. Through these studies, the authors suggested that multiple donors (even when using a triol) surround the fluorine atom of the benzyl fluoride, thus stabilising the transition state through substantial F···HOR hydrogen bond interactions, rather than through electrostatic stabilisation only [3]. This stabilisation was suggested to lead to a purely associative bimolecular (S_N_2) mechanism. The authors also studied the C–F activated Friedel–Crafts reactions [6–7] using very strong hydrogen bond donors, namely HFIP, in the presence or absence of trifluoroacetic acid (TFA). For both of these activators, Paquin et al. proposed a dissociative unimolecular (S_N_1) mechanism, whereby the strong hydrogen bond donor associates with the benzyl fluoride, leading to ionisation of the molecule, generating a benzylic carbocation and a formal equivalent of HF (which behaves in an autocatalytic manner as a stronger hydrogen bond donor than HFIP or TFA).

**Figure 2:**
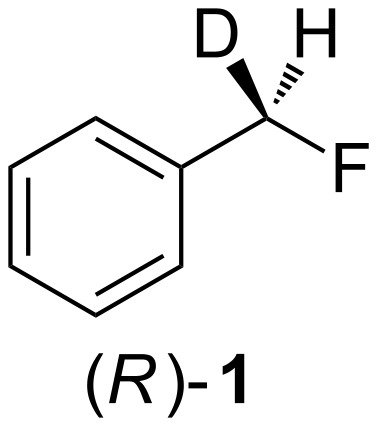
7-[^2^H_1_]-(*R*)-Benzyl fluoride ((*R*)-**1**).

Overall, there are three possible mechanistic pathways that these C–F activation reactions could follow: S_N_1, S_N_2, and a mixed S_N_1/S_N_2 pathway. Typically, benzylic substitutions would be expected to display a significant level of S_N_1 character. However, given the particularly poor properties of fluoride as a leaving group, developing a better understanding of the dissociative nature of these transformations remains of considerable interest. A direct bimolecular S_N_2 substitution would result in a complete inversion of configuration of the stereocenter and perfect enantiospecificity, while an S_N_1 mechanism would yield a fully racemized product. Any mixed pathway would generate products with partially racemized stereocenters. In this context, we decided to explore the stereointegrity of the aforementioned reactions using enantiopure 7-[^2^H_1_]-(*R*)-benzyl fluoride ((*R*)-**1,** Figure 2) as a primary, yet chiral electrophile [10].

Substitution reactions of benzyl fluoride (**1**) will generate substituted products that retain the deuterium atom, and the degree of stereointegrity can be determined by examining the enantiopurity of the isotopically labelled product. Quadrupolar ^2^H-nuclei can serve as a particularly useful NMR probe for assaying enantiopurity. If the ^2^H NMR is recorded in a lyotropic liquid crystalline solvent, where tumbling of the solute is restricted, then the ^2^H NMR signal splits into a doublet due to differential interactions of the quadrupolar nuclei with the electric field gradient associated with the oriented media [10]. When placed in an enantiomerically enriched liquid crystalline environment, the enantiomeric isotopomers interact unequally with the electric field gradients associated with the orientated media, creating anisotropy and resolving into two sets of doublets. If there is sufficient resolution between these quadrupolar couplings, then the enantiomeric ratio can be recorded. We have used poly-γ-benzyl-L-glutamate (PBLG) previously as the liquid-crystalline matrix for the determination of ee of samples of deuterated benzyl alcohols, benzyl fluorides, and esters of fluoroacetic acid [11] by ^2^H NMR and found it to be effective for the resolution of enantiomers. In this study, we explore various nucleophilic substitutions and a Friedel–Crafts reaction on enantiomerically labelled [^2^H_1_]benzyl fluoride.

## Results and Discussion

**Scheme 1:**
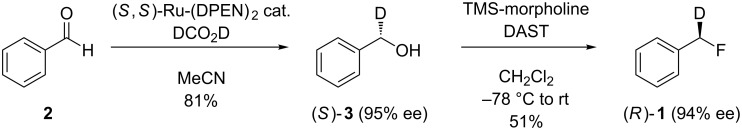
Synthesis of enantioenriched 7-[^2^H_1_]-(*R*)-benzyl fluoride ((*R*)-**1**) from benzaldehyde (**2**).

Highly enantiomerically enriched 7-[^2^H_1_]-(*R*)-benzyl fluoride ((*R*)-**1**) was synthesised in two steps from benzaldehyde (**2**), as described previously [11]; the procedure is summarised in Scheme 1.

Aldehyde **2** was reduced under Noyori’s conditions [12] using (*S*,*S*)-Ru(DPEN)_2_ as catalyst and [^2^H_2_]-formic acid as the deuterium source. This afforded the corresponding 7-[^2^H_1_]-(*S*)-benzyl alcohol ((*S*)-**3**) in moderate yield (81%) and high ee (95%), as evidenced by ^2^H-PBLG-NMR. Benzyl alcohol **3** was converted to the corresponding benzyl fluoride (**1**) using a modification of Bio’s method [13–14] to promote the S_N_2 reaction exclusively, using TMS-morpholine and DAST, in moderate yield (51%) and high ee (94%).

**Figure 3:**
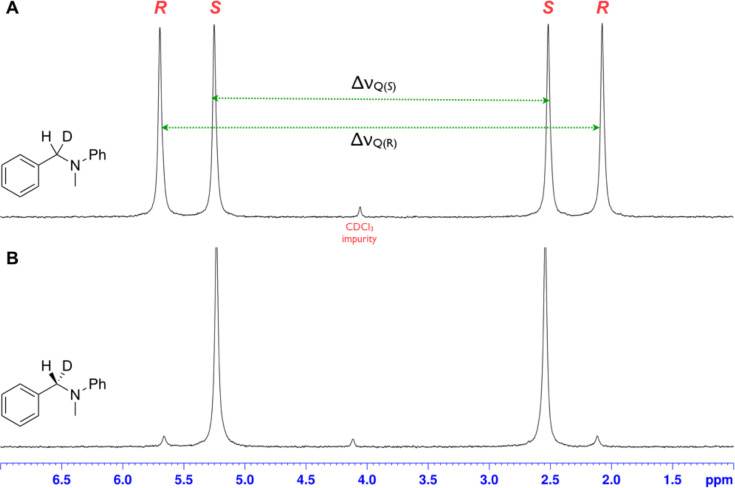
Partial ^2^H{^1^H} NMR (107.5 MHz) with PBLG in CHCl_3_ (13% w/w). (A) racemic sample of **6** (from Table 1, entry 2) and (B) enantioenriched sample of **6** (from Table 2, entry 2). The magnitude of the quadrupolar splittings for the (*R*)- and (*S*)-enantiomers are labelled Δν_Q(ent)_. The ee of each sample was determined by deconvolution of the line shapes and subsequent integration.

The isotopically enriched [^2^H_1_]-benzyl fluoride ((*R*)-**1**, 95% ee) was then subjected to a range of C–F activation reactions using a mixture of nucleophiles (for direct substitutions) and aryls (for Friedel–Crafts reactions) to give products **5**–**9**. The nucleophilic substitution reactions of **1** are shown in Table 1 and Table 2, and were all conducted using either a mixture of water/isopropanol, or tris(hydroxymethyl)propane as the activating hydrogen bond donor. In addition, three reactions of racemic substrates (Table 1, entries 1–3), were performed in order to ensure that sufficient resolution could be obtained in the ^2^H{^1^H}-PBLG-NMR, therefore allowing the ee of the products to be determined. A representative example of the ^2^H NMR spectra (107.5 MHz) is displayed in Figure 3, using *N*-methylaniline as a nucleophile, showing the spectra of both a racemic sample (Figure 3A) and an enantioenriched sample (Figure 3B) of **6**. However, as evidenced by entry 3 (Table 1), and entries 3 and 7 (Table 2), the analysis revealed that some nucleophiles (such as *N*-methylbenzylamine and morpholine [not shown]) were unsuitable for this study, as the resulting products **7** could not be resolved in the ^2^H NMR with PBLG assay.

**Table 1:** Nucleophilic substitution reactions of racemic 7-[^2^H_1_]-benzyl bromide (**4**).

Entry	Reaction	ee (%)

1	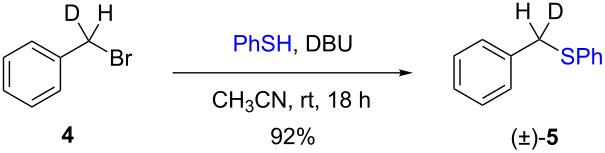	racemic
2	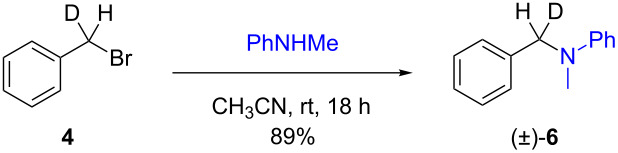	racemic
3	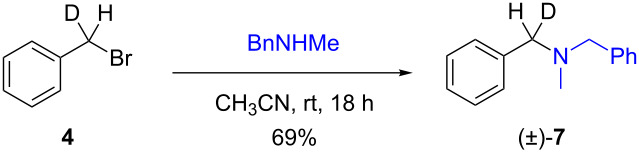	nd^a^

^a^ee could not be determined as a result of poor ^2^H{^1^H} NMR resolution.

**Table 2:** Nucleophilic substitution reactions of 7-[^2^H_1_]-(*R*)-benzyl fluoride ((*R*)-**1**).

Entry	Reaction	ee (%)

1	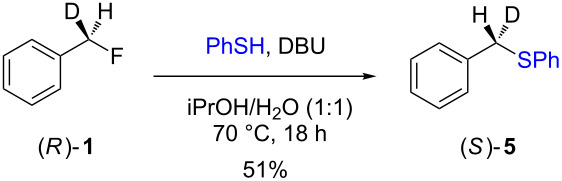	94
2	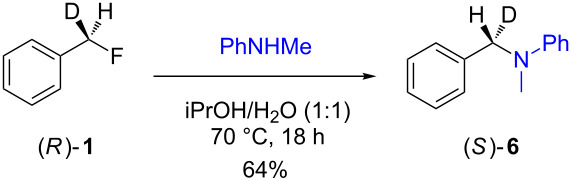	90
3	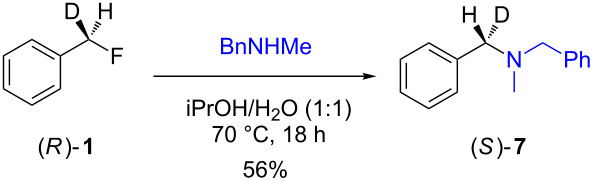	nd^a^
4	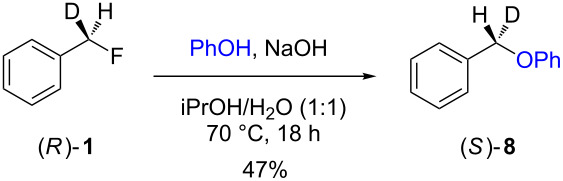	93
5	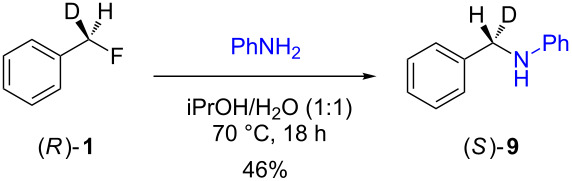	91
6	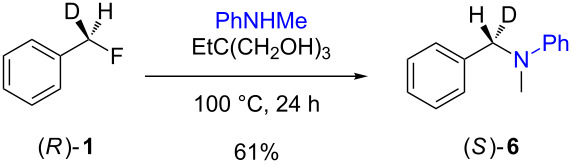	87
7	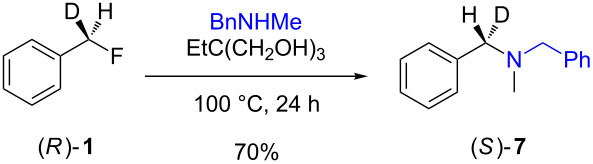	nd^a^
8	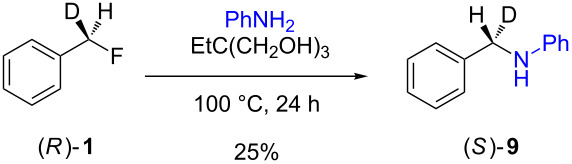	89

^a^ee could not be determined as a result of poor ^2^H{^1^H} NMR resolution.

Two different activator systems were investigated for the nucleophilic substitution of **1**: a mixture of water and isopropanol (Table 2, entries 1–5) and tris(hydroxymethyl)propane (Table 2, entries 6–8). Using water/isopropanol as the activator afforded the benzylated products **5**–**9** in moderate yields after 18 h. The ee values of all of the resulting products was very close to that of the original benzyl fluoride ((*R*)-**1**, 95%), indicating that a highly associative S_N_2-like pathway was operating, where the incoming nucleophile must have approached on a coordinate anti to the C–F bond resulting in an inversion of the configuration. These results are in good agreement with the transition state proposed by Paquin [3–5]. Unfortunately, *N*-methylbenzylamine (Table 2, entry 3) afforded a product **7** that did not resolve by ^2^H NMR, and thus the ee could not be determined.

Changing the activator from water/isopropanol to tris(hydroxymethyl)propane was anticipated to increase the stability of the triol–benzyl fluoride complex, and hence a tendancy towards an associative mechanism was expected. However, on performing the reactions with nitrogen nucleophiles (Table 2, entries 6 and 8) and the triol as the hydrogen bond donor, slightly lower ee’s were obtained relative to those obtained using the same nucleophiles with the water/isopropanol system (Table 2, entries 2 and 5). These minor differences in ee may be due to the higher temperature leading to a minor, but noticeable dissociative pathway. Once again, using *N*-methylbenzylamine as the nucleophile (Table 2, entry 7) afforded **7**, which could not be resolved by ^2^H NMR. Overall, the nucleophilic substitution of **1**, using either of the described hydrogen bond activating systems, afforded enantioenriched benzylated products with little erosion in stereointegrity.

In contrast to the above nucleophilic substitutions, which all proceeded with good stereointegrity, the Friedel–Crafts reactions of **1** with *p*-xylene gave very different results, as shown in Table 3.

**Table 3:** Friedel–Crafts reactions of 7-[^2^H_1_]-(*R*)-benzyl fluoride ((*R*)-**1**).

Entry	Reaction	ee (%)

1	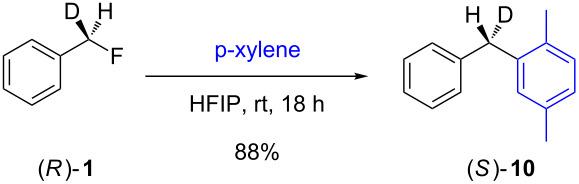	24
2	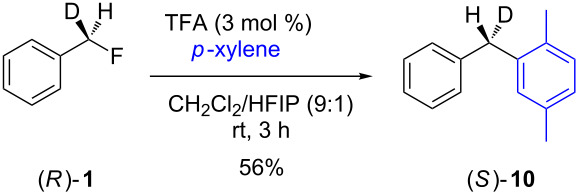	19
3	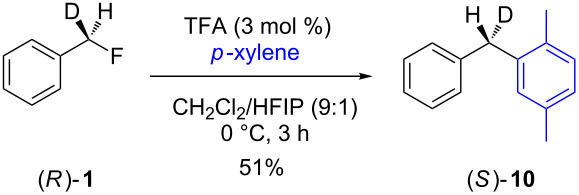	28

**Scheme 2:**
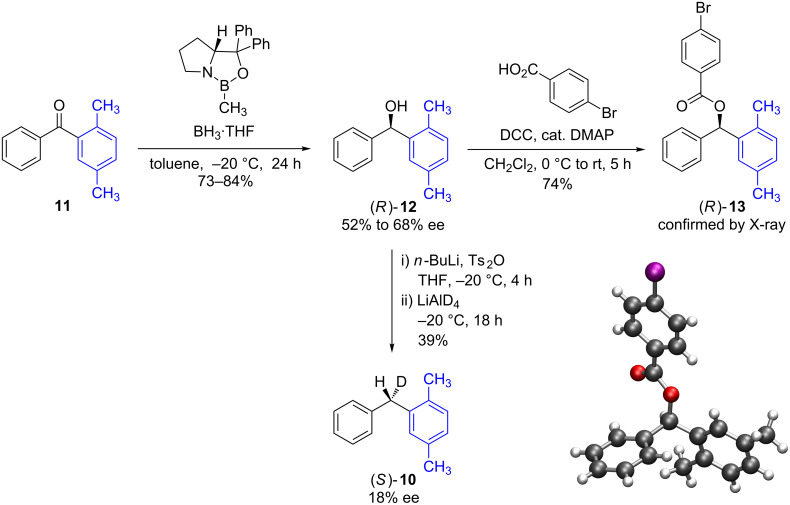
Synthesis of enantioenriched (*S*)-diarylmethane **10** from diaryl ketone **11** and confirmation of configuration of (*R*)-**13** by single crystal X-ray structure.

At room temperature (Table 3, entry 1), benzyl fluoride (*R*)-**1** was activated by HFIP, affording biarylmethane **10** in a good yield (88%) after 18 h. The ee of the product was low (24%), but not racemic. The proposed stereochemistry of the product was verified by independent synthesis of the (*S*)-isomer from the unsymmetric diphenyl ketone **11** (Scheme 2).

Corey–Bakshi–Shibata reduction of diaryl ketone **11**, afforded the (*R*)-alcohol **12** in moderate to good yield and moderate ee [15–16]. The absolute stereochemistry of **12** was confirmed by X-ray crystallography of the 4-bromophenyl ester derivative **13**. Alcohol **12** was activated as the tosyl ester at −20 °C, and then immediately displaced by LiAlD_4_ [17], inverting the stereocenter to afford the (*S*)-diarylmethane **10** isotopomer in 18% ee. ^2^H{^1^H} NMR in a PBLG matrix indicated that the dominant isomer was the same as was produced in entry 1, Table 3. Therefore, this analysis showed that the dominant enantiomer of **10** arose from an inversion, rather than retention, of configuration of the original stereocenter of **1**.

**Figure 4:**
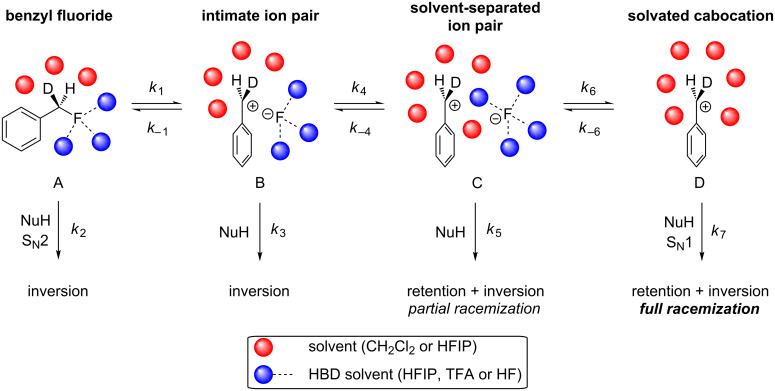
Possible reactive intermediates for C–F activation of benzyl fluoride **1** with strong hydrogen bond donors.

There may be four different reaction mechanisms operating in these Friedel–Crafts reactions as shown in Figure 4. (A) Coordination of the fluorine atom with the hydrogen bond donor, followed by backside attack of the nucleophile leads to S_N_2 reaction and inversion of configuration. (B) Hydrogen bond donor coordination to fluorine leads to ionisation of **1**, producing an intimate ion pair, which only permits backside attack of the nucleophile on the benzylic cation. (C) If the nucleophile is poor and *k*_4_ > *k*_3_, a solvent-separated ion pair will be formed, where the HBD-coordinated fluorine atom is loosely associated with the solvated cation, allowing a nucelophilic attack to occur from more trajectories, leading to a mixture of inversion (predominant) and retention products. (D) Fully solvated cation, where attack of the nucleophile can freely occur from either face, leading to racemization of the product in an S_N_1 reaction.

We propose that the actual attack of the nucleophile does not occur on the coordinated benzyl fluoride (A)**,** or the fully solvated carbocation (D), as these scenarios would incur 100% or 0% enantiospecificity, respectively. Rather, the data suggest that attack occurs on a mixture of intimate (B) and solvent-separated (C) ion pairs. The partial racemization observed in Table 3 suggests that the solvent-separated ion-pair intermediate (C) is most likely the reactive species, as it would naturally lead to a partial racemization of the substrate stereocenter.

When the activator was changed from HFIP to a mixed system of HFIP and TFA (3 mol %, Table 3, entries 2 and 3), the reactions were complete in a significantly shorter time, i.e., the initial induction period observed when only HFIP was used [7] disappeared in each case. The ee of entry 2 was lower (19%), showing that the increased hydrogen bonding strength of the TFA, and thus the more rapid generation of HF (vide infra), promotes a dissociative pathway via the solvent-separated ion pair. The greater ionic strength of the solution may also play a part in stabilising the partially dissociated carbocation. Pleasingly, decreasing the temperature (Table 3, entry 3) did not slow the reaction down, and it completed after 3 h. However, the decreased temperature lead to a slightly higher ee (28%) for **10**, suggesting that at lower temperatures the separation of the ions is less favoured in solution, presumably for entropic reasons.

The nature of the poorer nucleophile, coupled with the stronger hydrogen bond donor in the Friedel–Crafts reaction allows the solvent-separated ion-pair mechanism to predominate, significantly eroding the stereointegrity of the biarylmethane products **10**. However, the products were not racemic, showing that the nucleophilic attack also occurs via an associated ion pair, rather than the fully solvated carbocation.

## Conclusion

In summary, we have analyzed the stereochemical outcomes of substitution and Friedel–Crafts reactions of 7-[^2^H_1_]-(*R*)-benzyl fluoride ((*R*)-**1**), mediated by C–F activation using hydrogen-bond donors. When strong nucleophiles are used in conjunction with hydroxyl-based donors, an associative S_N_2-like reaction mechanism predominates, with almost complete inversion of the configuration at the stereogenic center. Poorer aryl nucleophiles can be used for Friedel–Crafts reactions if strong hydrogen bond donors (such as HFIP or TFA) are used to activate the C–F bond. In these cases, a dissociative mechanism operates, probably via a solvent-separated ion pair, rather than a fully solvated benzylic carbocation. The products arising from this mechanism are only partially enantioenriched, suggesting that there is still a steric influence for backside attack of the nucleophile in the solvent-separated ion pair, arising from the large, congested hydrogen bond networks around the fluorine atom.

## Supporting Information

The Supporting Information features experimental protocols and ^1^H, ^19^F (where appropriate) and ^2^H{^1^H} NMR spectra of benzyl fluoride **1** and adducts **5–10**. The methods for measurement of the ee by ^2^H{^1^H} NMR are also described.

File 1 Experimental protocols.

File 2 ^2^H NMR analysis of enantiopurity.
