# Supplementary material for: Stereochemical outcomes of C–F activation reactions of benzyl fluoride
Source: Beilstein J Org Chem. 2018 Jan 9;14:106–13. doi: 10.3762/bjoc.14.6 (PMC5789430; doi:10.3762/bjoc.14.6)
Supplement: File 1 — Experimental protocols. [file Beilstein_J_Org_Chem-14-106-s001.pdf]

Supporting Information File 1

for

Stereochemical outcomes of C–F activation reactions of benzyl fluoride

Neil S. Keddie[†,1], Pier Alexandre Champagne[†,2], Justine Desroches[2], Jean-François Paquin*[,2] and David O'Hagan*[,1]

Address: [1]School of Chemistry, Biomedical Sciences Research Complex, University of St Andrews, North Haugh, St Andrews, Fife KY16 9ST, United Kingdom and [2]PROTEO, CCVC, Département de chimie, 1045 Avenue de la Médecine, Université Laval, Québec, QC G1V 0A6, Canada

Email: Jean-François Paquin - jean-francois.paquin@chm.ulaval.ca; David O'Hagan - do1@st-andrews.ac.uk

[†] These authors contributed equally to this work.

* Corresponding author

Experimental protocols

1. General information

Unless otherwise noted, all commercial reagents were used without further purification. Dichloromethane and acetonitrile were purified using a Vacuum Atmospheres Inc. Solvent Purification System. Thin-layer chromatography (TLC) analysis of reaction mixtures was performed using Silicyle silica gel 60 Å F_{254} TLC plates, and visualized under UV or by staining with iodine. Flash column chromatography was carried out on Silicycle Silica Gel 60 Å, 230 × 400 mesh. High resolution mass spectra were obtained on a LC/MS–TOF Agilent 6210 using either electrospray ionization (ESI) or atmospheric pressure photoionization (APPI). ^{1}H and ^{13}C nuclear magnetic resonance (NMR) spectra were recorded using Agilent DD2 500 and Varian Inova 400 spectrometers. ^{1}H and ^{13}C chemical shifts are reported in ppm downfield of tetramethylsilane and referenced to tetramethylsilane (δ = 0.00 ppm) or residual chloroform peak (δ = 7.26 ppm). Coupling constants (*J*) are measured in hertz (Hz). Multiplicities are reported using the following abbreviations: s = singlet, d = doublet, t = triplet, q = quartet, m = multiplet, br = broad resonance. Infrared spectra were recorded using a Thermo Scientific Nicolet 380 FTIR spectrometer. Melting points were recorded on a Stanford Research System OptiMelt capillary melting point apparatus and are uncorrected.

2. Experimental data

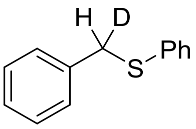

7-[$^{2}H_{1}$]Benzyl phenyl thioether (5)

^{1}H NMR (500 MHz, $CDCl_3$) δ (ppm) = 7.40 – 7.27 (m, 9H), 7.27 – 7.21 (m, 2H), 4.16 (t, *J* = 1.8 Hz, 1H); ^{13}C NMR (126 MHz, $CDCl_3$) δ (ppm) =137.5, 136.5, 129.9, 128.97, 128.95, 128.6, 127.3, 126.5, 38.9 (t, *J* = 21.5 Hz); IR (ATR, ZnSe) ν (cm^{-1}) = 3059, 2922, 1583, 1569, 1478, 1451, 1438, 1021, 729, 699, 685; HRMS-ESI (+) *m/z* calcd for $C_{13}H_{11}DNaS$ $[M+Na]^{+}$ 224.0615 found 224.0639.

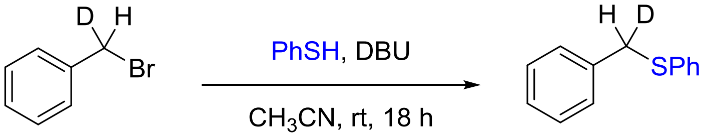

4 92% **(±)-5**

To a stirred solution of 7-[$^{2}H_{1}$]benzyl bromide (75 mg, 0.436 mmol, 1.0 equiv) under argon atmosphere in dry CH_3CN (1.8 mL) were added thiophenol (90 µL, 2.0 equiv)

and 1,8-diazabicyclo[5.4.0]undec-7-ene (126 µL, 1.9 equiv). The resulting solution was stirred at room temperature for 18 h. The reaction was quenched with 1 M NaOH and extracted with Et_2O (3×). The combined organic extracts were washed with 1 M NaOH, 3 M HCl and H_2O, dried with $MgSO_4$, filtered and evaporated under reduced pressure. The crude product was purified by silica gel chromatography using hexanes as the eluent to yield (±)-7-[$^2H_1$]benzyl phenyl thioether **5** (81 mg, 92%) as a colorless solid; ee was racemic by $^2H\{^1H\}$ NMR analysis with PBLG in $CHCl_3$.

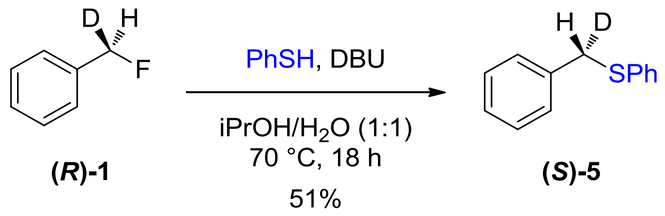

(***R***)**-1** (***S***)**-5**

In a sealable vial, were successively added thiophenol (220 µL, 3.0 equiv), 1,8-diazabicyclo[5.4.0]undec-7-ene (323 µL, 3.0 equiv), isopropanol (0.72 mL) and H_2O (0.72 mL). (*R*)-7-[$^2H_1$]benzyl fluoride ((*R*)-**1**, 75 mg, 0.436 mmol, 1.0 equiv) was then added and the vial was sealed. The resulting solution was stirred at 70 °C for 18 h. The reaction was quenched with $NaHCO_3$ aq. sat. and extracted with Et_2O (3×). The combined organic extracts were washed with brine, dried with $MgSO_4$, filtered and evaporated under reduced pressure. The crude product was purified by silica gel chromatography using hexanes/ethyl acetate (99/1) as the eluent to yield (*S*)-7-[$^2H_1$]-benzyl phenyl thioether **5** (73 mg, 51%) as a colorless solid; 94% ee by $^2H\{^1H\}$ NMR analysis with PBLG in $CHCl_3$.

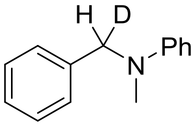

***N*-(7-[$^2H_1$]Benzyl)-*N*-methylaniline (6)**

^1H NMR (500 MHz, $CDCl_3$) δ (ppm) = 7.37 – 7.33 (m, 2H), 7.30 – 7.23 (m, 5H), 6.81 – 6.77 (m, 2H), 6.75 (tq, *J* = 7.2, 1.1 Hz, 1H), 4.55 (t, *J* = 2.3 Hz, 1H), 3.05 (s, 3H); ^{13}C NMR (126 MHz, $CDCl_3$) δ (ppm) = 149.8, 139.1, 129.3, 128.7, 127.0, 126.9, 116.6, 112.4, 56.4 (t, *J* = 20.1 Hz), 38.6; IR (ATR, ZnSe) ν (cm^{-1}) = 3026, 2897, 1506, 1452, 1370, 1031, 861, 730; HRMS-ESI (+) *m/z* calcd for $C_{14}H_{14}DNNa$ $[M+Na]^+$ 221.1160 found 221.1162.

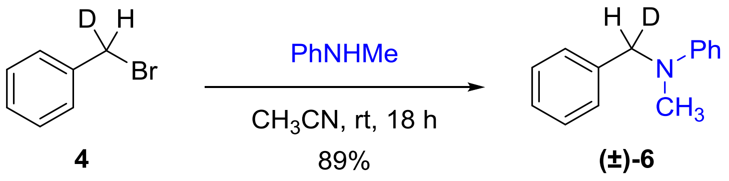

To a stirred solution of 7-[$^2H_1$]benzyl bromide (75 mg, 0.436 mmol, 1.0 equiv) under argon atmosphere in dry CH_3CN (2.0 mL) was added *N*-methylaniline (142 µL, 3.0 equiv). The resulting solution was stirred at room temperature for 18 h. The reaction was quenched with $NaHCO_3$ aq. sat. and extracted with Et_2O (3×). The combined organic extracts were washed with H_2O, dried with $MgSO_4$, filtered and evaporated under reduced pressure. The crude product was purified by silica gel chromatography using hexanes/ethyl acetate (100:0 to 98:2) as the eluent to yield (±)-*N*-(7-[$^2H_1$]-benzyl)-*N*-methylaniline **6** (77 mg, 89%) as a yellow oil; ee was racemic by $^2H\{^1H\}$ NMR analysis with PBLG in $CHCl_3$.

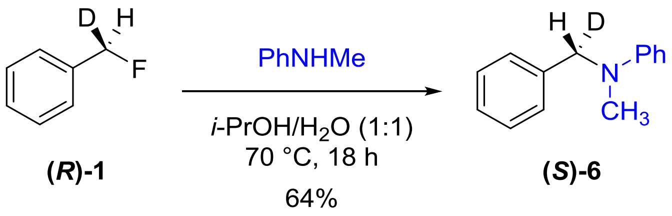

In a sealable vial, were successively added (*R*)-7-[$^2H_1$]benzyl fluoride (83 mg, 0.747 mmol, 1.0 equiv), isopropanol (0.75 mL), H_2O (0.75 mL) and *N*-methylaniline (243 µL, 3.0 equiv). The vial was sealed and the resulting solution was stirred at 70 °C for 18 h. The reaction was quenched with 1 M Na_2CO_3 and extracted with Et_2O (3×). The combined organic extracts were washed with brine, dried with $MgSO_4$, filtered and evaporated under reduced pressure. The crude product was purified by silica gel chromatography using hexanes/ethyl acetate (100:0 to 99:1) as the eluent to yield (*S*)-*N*-(7-[$^2H_1$]-benzyl)-*N*-methylaniline ((*S*)-**6**, 56 mg, 64%) as a yellow oil; 90% ee by $^2H\{^1H\}$ NMR analysis with PBLG in $CHCl_3$.

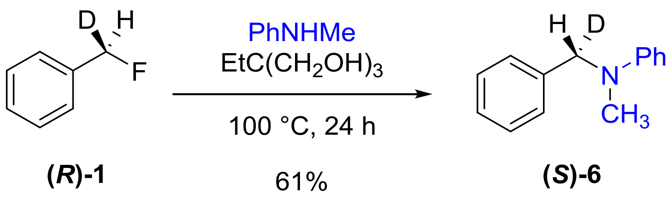

In a sealable vial, under argon atmosphere, were successively added (*R*)-7-[$^2H_1$]-benzyl fluoride (81 mg, 0.720 mmol, 1.0 equiv), freshly ground 1,1,1-

tris(hydroxymethyl)propane (106 mg, 1.1 equiv) and *N*-methylaniline (153 µL, 2.0 equiv). The vial was sealed and the resulting solution was stirred at 100 °C for 24 h. The reaction was quenched with 1 M Na_2CO_3 and extracted with Et_2O (3×). The combined organic extracts were washed with brine, dried with $MgSO_4$, filtered and evaporated under reduced pressure. The crude product was purified by silica gel chromatography using hexanes/ethyl acetate (98:2) as the eluent to yield (*S*)-*N*-(7-[$^2H_1$]benzyl)-*N*-methylaniline ((*S*)-**6**, 53 mg, 61%) as a yellow oil; 87% ee by $^2H\{^1H\}$ NMR analysis with PBLG in $CHCl_3$.

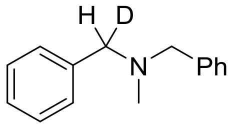

N-(7-[$^2H_1$]Benzyl)-N-methyl-1-phenylmethanamine (7)

^1H NMR (500 MHz, $CDCl_3$) δ (ppm) = 7.42 – 7.37 (m, 4H), 7.37 – 7.32 (m, 4H), 7.29 – 7.24 (m, 2H), 3.56 – 3.54 (m, 2H), 3.51 (br s, 1H), 2.21 (s, 3H); ^{13}C NMR (126 MHz, $CDCl_3$) δ (ppm) = 139.5, 139.4, 129.07, 129.05, 128.3, 127.05, 127.04, 62.0, 61.6 (t, *J* = 20.1 Hz), 42.4; IR (ATR, ZnSe) ν (cm^{-1}) = 3028, 2790, 1494, 1453, 1363, 1024, 736, 698; HRMS-ESI (+) *m/z* calcd for $C_{15}H_{16}DNNa$ $[M+Na]^+$ 235.1316, found 235.1309.

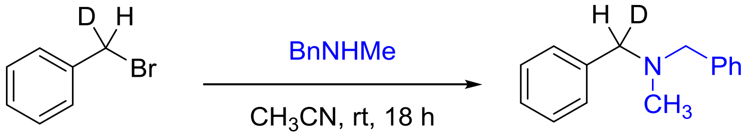

4 **(±)-7**

To a stirred solution of 7-[$^2H_1$]benzyl bromide (75 mg, 0.436 mmol, 1.0 equiv) under argon atmosphere in dry CH_3CN (2.0 mL) was added *N*-benzylmethylamine (170 µL, 3.0 equiv). The resulting solution was stirred at room temperature for 18 h. The reaction was quenched with 1 M Na_2CO_3 and extracted with Et_2O (3×). The combined organic extracts were washed with H_2O, dried with $MgSO_4$, filtered and evaporated under reduced pressure. The crude product was purified by silica gel chromatography using hexanes/ethyl acetate (98/2) as the eluent to yield (±)-*N*-(7-[$^2H_1$]benzyl)-*N*-methyl-1-phenylmethanamine (**7**, 64 mg, 69%) as a pale yellow oil; ee could not be determined as a result of poor $^2H\{^1H\}$ NMR resolution.

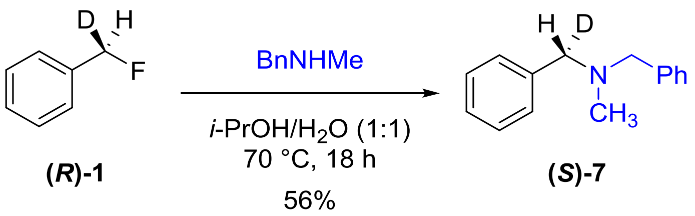

In a sealable vial, were successively added (*R*)-7-[$^2H_1$]benzyl fluoride (80 mg, 0.720 mmol, 1.0 equiv), isopropanol (0.72 mL), H_2O (0.72 mL) and *N*-benzylmethylamine (279 µL, 3.0 equiv). The vial was sealed and the resulting solution was stirred at 70 °C for 18 h. The reaction was quenched with 1 M Na_2CO_3 and extracted with Et_2O (3×). The combined organic extracts were washed with brine, dried with $MgSO_4$, filtered and evaporated under reduced pressure. The crude product was purified by silica gel chromatography using hexanes/ethyl acetate (98/2) as the eluent to yield (*S*)-*N*-(7-[$^2H_1$]benzyl)-*N*-methyl-1-phenylmethanamine ((*S*)-**7**, 85 mg, 56%) as a colorless oil; ee could not be determined as a result of poor $^2H\{^1H\}$ NMR resolution

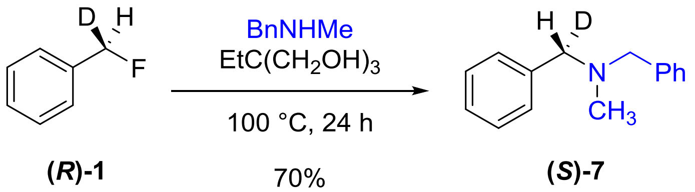

In a sealable vial, under argon atmosphere, were successively added (*R*)-7-[$^2H_1$]-benzyl fluoride (80 mg, 0.720 mmol, 1.0 equiv), freshly ground 1,1,1-tris(hydroxymethyl)propane (106 mg, 1.1 equiv) and *N*-benzylmethylamine (186 µL, 2.0 equiv). The vial was sealed and the resulting solution was stirred at 100 °C for 24 h. The reaction was quenched with 1 M Na_2CO_3 and extracted with Et_2O (3×). The combined organic extracts were washed with brine, dried with $MgSO_4$, filtered and evaporated under reduced pressure. The crude product was purified by silica gel chromatography using hexanes/ethyl acetate (98:2) as the eluent to yield (*S*)-*N*-(7-[$^2H_1$]benzyl)-*N*-methyl-1-phenylmethanamine ((*S*)-**7**, 108 mg, 70%) as a colorless oil; ee could not be determined as a result of poor $^2H\{^1H\}$ NMR resolution

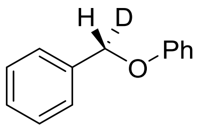

(*S*)-7-[$^2H_1$]Benzyl phenyl ether (8)

^1H NMR (500 MHz, $CDCl_3$) δ (ppm) = 7.46 – 7.42 (m, 2H), 7.42 – 7.35 (m, 2H), 7.35 – 7.27 (m, 3H), 7.01 – 6.93 (m, 3H), 5.06 (br s,

1H). Analytical data were identical to those previously reported [1].

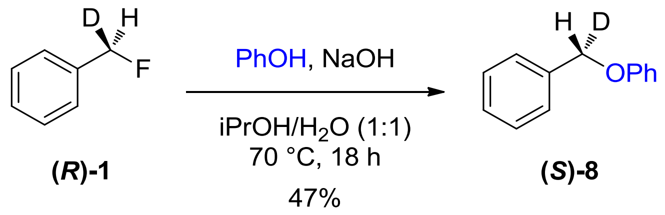

In a sealable vial, were successively added phenol (203 mg, 3.0 equiv), NaOH (86 mg, 3.0 equiv), isopropanol (0.72 mL) and H_2O (0.72 mL). (*R*)-7-[$^2H_1$]benzyl fluoride (80 mg, 0.720 mmol, 1.0 equiv) was then added and the vial was sealed. The resulting solution was stirred at 70 °C for 18 h. The reaction was quenched with 1M Na_2CO_3 and extracted with Et_2O (3×). The combined organic extracts were washed with brine, dried with $MgSO_4$, filtered and evaporated under reduced pressure. The crude product was purified by silica gel chromatography using hexanes/ethyl acetate (99:1) as the eluent to yield (*S*)-7-[$^2H_1$]-benzyl phenyl ether ((*S*)-**8**, 63 mg, 47%) as a colorless solid; 93% ee by $^2H\{^1H\}$ NMR analysis with PBLG in $CHCl_3$.

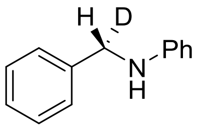

(*S*)-*N*-(7-[$^2H_1$]benzyl)aniline (9)

^1H NMR (500 MHz, $CDCl_3$) δ (ppm) = 7.40 – 7.33 (m, 4H), 7.31 – 7.26 (m, 11H), 7.20 – 7.15 (m, 2H), 6.72 (tt, *J* = 7.3, 1.1 Hz, 1H), 6.66 – 6.63 (m, 2H), 4.32 (t, *J* = 2.1 Hz, 1H), 4.03 (br s, 1H); ^{13}C NMR (126 MHz, $CDCl_3$) δ (ppm) = 148.3, 139.5, 129.4, 128.8, 127.7, 127.4, 117.7, 113.0, 48.1 (t, *J* = 21.0 Hz); IR (ATR, ZnSe) ν (cm^{-1}) = 3416, 3024, 1600, 1503, 1312, 746, 718, 689; HRMS-ESI (+) *m/z* calcd for $C_{13}H_{13}DN$ $[M+H]^+$ 185.1184, found 185.1182.

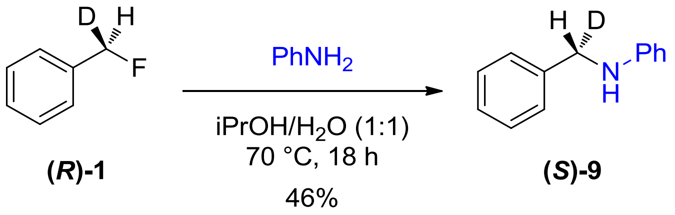

In a sealable vial, were successively added (*R*)-7-[$^2H_1$]benzyl fluoride (80 mg, 0.720 mmol, 1.0 equiv), isopropanol (0.72 mL), H_2O (0.72 mL) and aniline (197 µL, 3.0 equiv). The vial was sealed and the resulting solution was stirred at 70 °C for

18 h. The reaction was quenched with 1M Na_2CO_3 and extracted with Et_2O (3×). The combined organic extracts were washed with brine, dried with $MgSO_4$, filtered and evaporated under reduced pressure. The crude product was purified by silica gel chromatography using hexanes/ethyl acetate (98:2) as the eluent to yield (*S*)-*N*-(7-[$^2H_1$]benzyl)aniline ((*S*)-**9**, 61 mg, 46%) as a yellow oil; 91% ee by $^2H\{^1H\}$ NMR analysis with PBLG in $CHCl_3$.

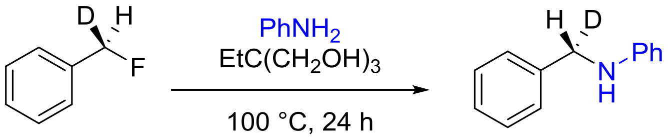

In a sealable vial, under argon atmosphere, were successively added (*R*)-7-[$^2H_1$]-benzyl fluoride (80 mg, 0.720 mmol, 1.0 equiv), freshly ground 1,1,1-tris(hydroxymethyl)propane (106 mg, 1.1 equiv) and aniline (131 µL, 2.0 equiv). The vial was sealed and the resulting solution was stirred at 100 °C for 24 h. The reaction was quenched with 1 M Na_2CO_3 and extracted with Et_2O (3×). The combined organic extracts were washed with brine, dried with $MgSO_4$, filtered and evaporated under reduced pressure. The crude product was purified by silica gel chromatography using hexanes/ethyl acetate (98:2) as the eluent to yield (*S*)-*N*-(7-[$^2H_1$]benzyl)-*N*-methyl-1-phenylmethanamine ((*S*)-**9**, 33 mg, 25%) as a yellow oil; 89% ee by $^2H\{^1H\}$ NMR analysis with PBLG in $CHCl_3$.

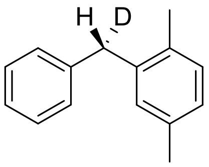

(*S*)-2-(7-[$^2H_1$]Benzyl)-1,4-dimethylbenzene (10)

^{1}H NMR (500 MHz, $CDCl_3$) δ (ppm) = 7.32 – 7.26 (m, 2H), 7.23 – 7.18 (m, 1H), 7.17 – 7.13 (m, 2H), 7.08 (d, *J* = 7.6 Hz, 1H), 6.99 (d, *J* = 7.6 Hz, 1H), 6.96 (s, 1H), 3.97 – 3.94 (m, 1H), 2.31 (s, 3H), 2.22 (s, 3H); ^{13}C NMR (101 MHz, $CDCl_3$) δ (ppm) = 140.6, 138.8, 135.49, 135.48, 133.6, 130.9, 130.32, 130.31, 128.83, 128.5, 127.23, 127.22, 125.99, 125.98, 39.2 (t, *J* = 19.1 Hz), 21.1, 19.3; IR (ATR, ZnSe) ν (cm^{-1}) = 3024, 2920, 1493, 1449, 1030, 809, 697; HRMS-APPI *m/z* calcd for $C_{15}H_{15}D$ [M*]+ 197.1309, found 197.1310.

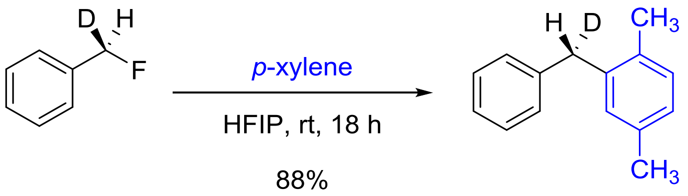

To a sealable vial were successively added (*R*)-7-[$^2H_1$]-benzyl fluoride (100 mg, 0.900 mmol, 1.0 equiv), *p*-xylene (555 µL, 5.0 equiv) and HFIP (3.6 mL). The resulting solution was stirred for 18 h at room temperature. The reaction was quenched with H_2O and extracted with CH_2Cl_2 (3×). The combined organic extracts were washed with brine, dried with $MgSO_4$, filtered and evaporated under reduced pressure. The crude product was purified by silica gel chromatography using hexanes/ethyl acetate (99:1) as the eluent to yield (*S*)-2-(7-[$^2H_1$]benzyl)-1,4-dimethylbenzene ((*S*)-**10**, 156 mg, 88%) as a colorless oil; 24% ee by $^2H\{^1H\}$ NMR analysis with PBLG in $CHCl_3$.

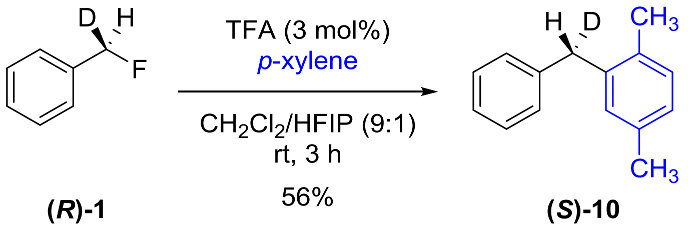

To a stirred solution of (*R*)-7-[$^2H_1$]benzyl fluoride (100 mg, 0.900 mmol, 1.0 equiv) in dry CH_2Cl_2 (90% of the volume required for substrate concentration of 0.25 M) were added *p*-xylene (555 µL, 5.0 equiv) and HFIP (10% of the volume required for substrate concentration of 0.25 M, resulting in a 9:1 mixture of CH_2Cl_2/HFIP). Finally, TFA (0.2 M in CH_2Cl_2, 5 mol % of TFA) was added. The resulting solution was stirred for 3 h at room temperature. The reaction was quenched with H_2O and extracted with CH_2Cl_2 (3×). The combined organic extracts were washed with brine, dried with $MgSO_4$, filtered and evaporated under reduced pressure. The crude product was purified by silica gel chromatography using hexanes/ethyl acetate (99:1) as the eluent to yield (*S*)-2-(7-[$^2H_1$]-benzyl)-1,4-dimethylbenzene ((*S*)-**10**, 100 mg, 56%) as a colorless oil; 19% ee by $^2H\{^1H\}$ NMR analysis with PBLG in $CHCl_3$.

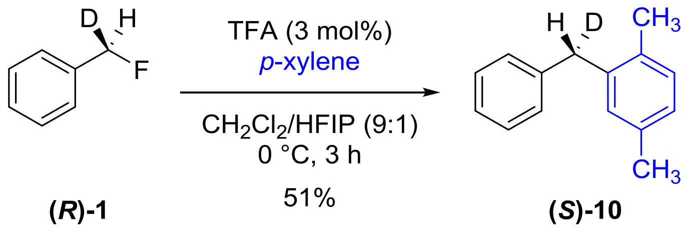

To a stirred solution of (*R*)-7-[$^2H_1$]benzyl fluoride (100 mg, 0.900 mmol, 1.0 equiv) at 0 °C in dry CH_2Cl_2 (90% of the volume required for substrate concentration of 0.25 M) were added *p*-xylene (555 µL, 5.0 equiv) and HFIP (10% of the volume required for substrate concentration of 0.25 M, resulting in a 9:1 mixture of CH_2Cl_2/HFIP). Finally, TFA (0.2 M in CH_2Cl_2, 5 mol % of TFA) was added. The resulting solution was stirred for 3 h at 0 °C. The reaction was quenched with H_2O and extracted with CH_2Cl_2 (3×). The combined organic extracts were washed with brine, dried with $MgSO_4$, filtered and evaporated under reduced pressure. The crude product was purified by silica gel chromatography using hexanes/ethyl acetate (99:1) as the eluent to yield (*S*)-2-(7-[$^2H_1$]-benzyl)-1,4-dimethylbenzene ((*S*)-**10**, 91 mg, 51%) as a colorless oil; 28% ee by $^2H\{^1H\}$ NMR analysis with PBLG in $CHCl_3$.

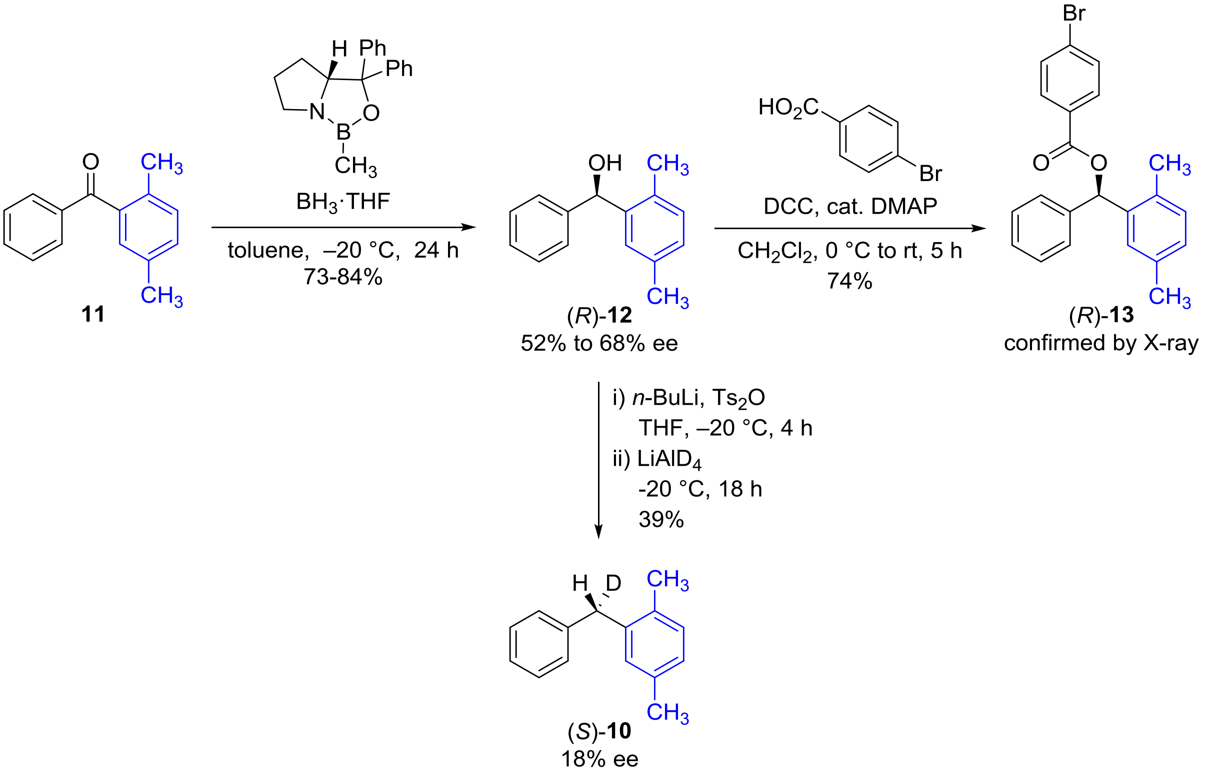

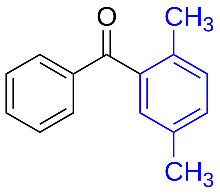

(2,5-Dimethylphenyl)(phenyl)methanone (11)

In a 50 mL round-bottomed flask with a magnetic stirrer, benzoyl chloride (826 µL, 7.11 mmol, 1 equiv) and *p*-xylene (1.75 mL, 14.22 mmol, equiv) were dissolved in 15 mL CH_2Cl_2. $AlCl_3$ (950 mg, 7.11 mmol, 1 equiv) was added and the reaction was stirred for 18 hours at 21 °C. The mixture was diluted with H_2O and additional CH_2Cl_2, extracted with CH_2Cl_2 (2×) and the organic phases were washed with H_2O (2×), brine, then dried over $MgSO_4$, filtered and evaporated. After silica gel chromatography using hexanes/EtOAc (97/3), the title compound (1.30 g, 87%) was isolated as a slightly orange liquid. Spectral data were identical to those previously reported [2].

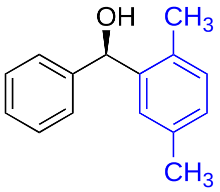

(*R*)-(2,5-Dimethylphenyl)(phenyl)methanol ((*R*)-12)

In a 100 mL round-bottomed flask equipped with a magnetic stir bar, (*R*)-2-methyl-CBS-oxazaborolidine (1 M in PhMe, 830 µL, 0.83 mmol, 0.15 equiv) was mixed with BH_3•THF (1 M in THF, 11.03 mL, 11.03 mmol, 2 equiv) at −20 °C. A solution of (2,5-dimethylphenyl)(phenyl)methanone (1,16 g, 5.52 mmol, 1 equiv) in PhMe (38 mL) was then slowly added at this temperature. The reaction was stirred at −20 °C for 18 h, and then the solvents were evaporated under reduced pressure. The crude mixture was diluted in CH_2Cl_2, washed with water, dried over Na_2SO_4, filtered and concentrated. After silica gel chromatography using hexanes/EtOAc (90/10), the title compound (989 mg, 84%) was isolated as a white solid. Spectral data were identical to those previously reported [2]. HPLC analysis (OJ-H, hexanes/iPrOH (95/5), 0.5 mL/min, 254.4 nm) t = 23.72 min (24.25%), 25.73 min (75.75%).

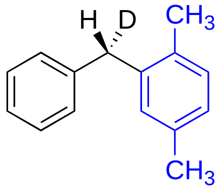

(*S*)-2-(7-[$^2H_1$]Benzyl)-1,4-dimethylbenzene ((*S*)-10)

In a glass vessel equipped with a magnetic stir bar, a solution of (*R*)-(2,5-dimethylphenyl)(phenyl)methanol (150 mg, 0.71 mmol, 1 equiv) in 3 mL THF was prepared under argon atmosphere, then cooled to −20 °C. *n*-BuLi (1.52 M in hexanes, 474 µL, 0.71 mmol, 1 equiv) was added dropwise and the mixture was stirred at −20 °C for one hour. Ts_2O (288 mg,

0.792 mmol, 1.1 equiv) was added and 3 mL THF was used to wash the vessel walls. The reaction was stirred at −20 °C for 4 h, then $LiAlD_4$ (90% purity, 66 mg, 1.44 mmol, 2 equiv) was added. The full mixture was stirred for 18 h at −20 °C, then quenched with NaOH (2 M). The slurry was extracted with CH_2Cl_2 (3×) and the combined organic extracts were washed with water, dried over $MgSO_4$, filtered and evaporated. Following silica gel chromatography using 100% hexanes, the pure product (54 mg, 39%) was isolated as a colorless liquid. Spectral data is as described above; $[\alpha]_D^{21}$ = -0.54 ° (c = 0.97, $CHCl_3$); 18% ee by $^2H\{^1H\}$ NMR analysis with PBLG in $CHCl_3$, as same enantiomer for **10** synthesised from (*R*)-**1** (See Supporting Information File 2 for spectrum).

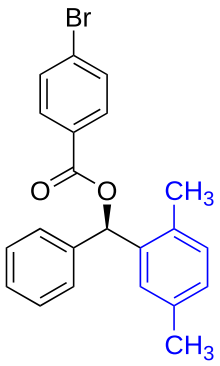

(*R*)-(2,5-dimethylphenyl)(phenyl)methyl 4-bromobenzoate ((*R*)-13)

In a glass vessel, 4-bromobenzoic acid (53 mg, 0.264 mmol, 1.1 equiv), DMAP (2 mg, 0.012 mmol, 5 mol %) and (*R*)-(2,5-dimethylphenyl)(phenyl)methanol (50 mg, 0.24 mmol, 1 equiv) were dissolved in 1 mL CH_2Cl_2. This solution was cooled to 0 °C and DCC (57 mg, 0.276 mmol, 1.15 equiv) was added. The reaction was stirred 5 min at 0 °C, then allowed back to room temperature and stirred for an additional 5 h. The reaction mixture was diluted with more CH_2Cl_2 and this organic phase was washed with HCl (10%, 2×), sat. $NaHCO_3$, and then H_2O. It was finally dried over $MgSO_4$, filtered and concentrated. The desired compound (70 mg, 74%) was isolated as white needles by column chromatography using hexanes/EtOAc (95/5). ^1H NMR (400 MHz, $CDCl_3$) δ 2.31 (s, 6H), 7.03-7.08 (m, 2H), 7.24 (m, 2H), 7.28-7.35 (m, 5H), 7.60 (d, *J* = 8.5 Hz, 2H), 7.98 (d, *J* = 8.5 Hz), 2H); ^{13}C NMR (100 MHz, $CDCl_3$) δ 19.1, 21.2, 75.3, 127.4, 127.7, 127.9, 128.3, 128.5, 128.9, 129.1, 130.7, 131.3, 131.8, 132.8, 135.6, 137.6, 139.3, 164.9; IR (ATR, ZnSe) ν = 2920, 1713, 1264, 1098, 1009, 812, 767, 704 cm^{-1}; HRMS-ESI calcd for $C_{22}H_{19}BrNaO_2$ $[M+Na]^+$ 417.0461, found 417.0441.

Crystal data and structure refinement for 13.

Empirical formula	$C_{22}H_{19}BrO_2$
Temperature	150(2) K
Wavelength	0.71073 Å
Crystal system	Orthorhombic
Space group	P 21 21 21
Unit cell dimensions	a = 7.1645(5) Å<br>b = 13.8680(10) Å<br>c = 18.0569(14) Å
Volume	1794.1(2) $Å^3$
Z	4
Density (calculated)	1.463 mg/m^3
F(000)	808
Crystal size	0.520 x 0.380 x 0.260 mm^3
Theta range for data collection	1.852 to 30.540°
Index ranges	-10<=h<=10, -19<=k<=19, -25<=l<=25
Reflections collected	23036
Independent reflections	5481 [R(int) = 0.0279]
Completeness to theta = 25.242°	100.0 %
Absorption correction	Semi-empirical from equivalents
Max. and min. transmission	0.549 and 0.364
Refinement method	Full-matrix least-squares on F2
Data / restraints / parameters	5481 / 0 / 228
Goodness-of-fit on F2	1.024
Final R indices [I>2sigma(I)]	R1 = 0.0269, wR2 = 0.0648
R indices (all data)	R1 = 0.0314, wR2 = 0.0663
Absolute structure parameter	0.011(3)
Largest diff. peak and hole	0.594 and -0.482 e.$Å^{-3}$

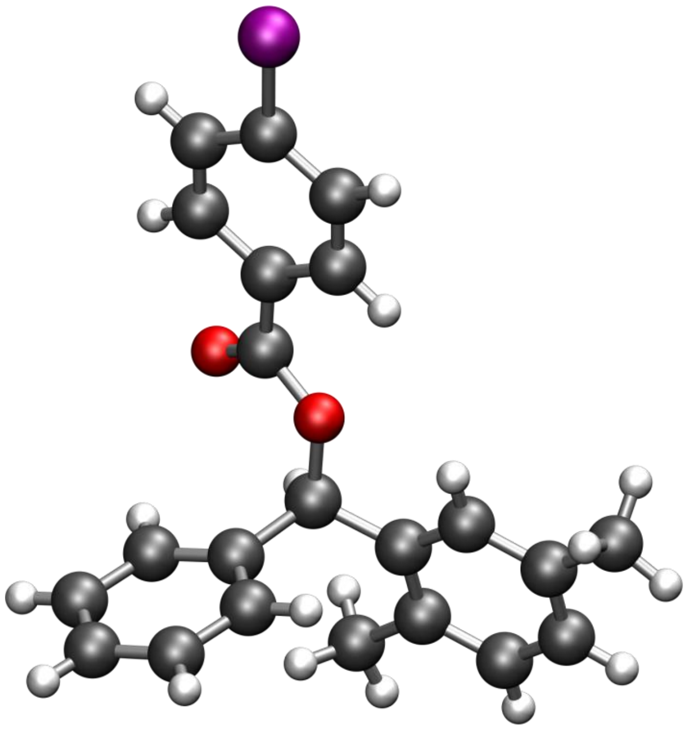

References

1. Velasco, R.; Feberero, C.; Sanz, R. *Org. Lett*. **2015**, *17*, 4416–4419.

2. Desroches, J.; Champagne, P. A.; Benhassine, Y.; Paquin, J.-F. *Org. Biomol. Chem.* **2015**, *13*, 2243.

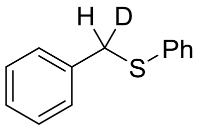

^{1}H NMR (400 MHz, $CDCl_3$)

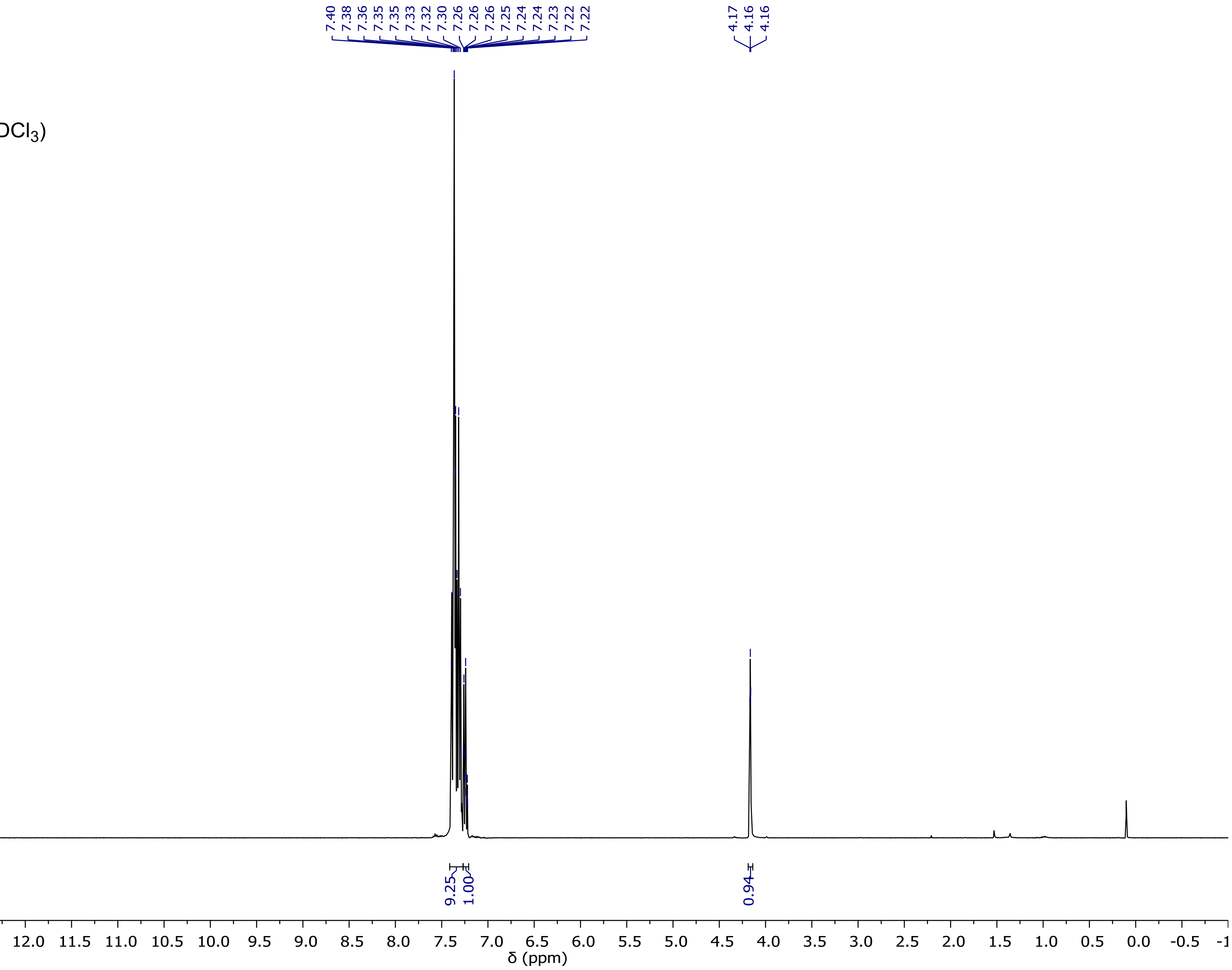

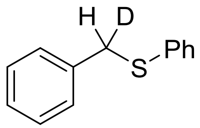

^{13}C NMR (126 MHz, $CDCl_3$)

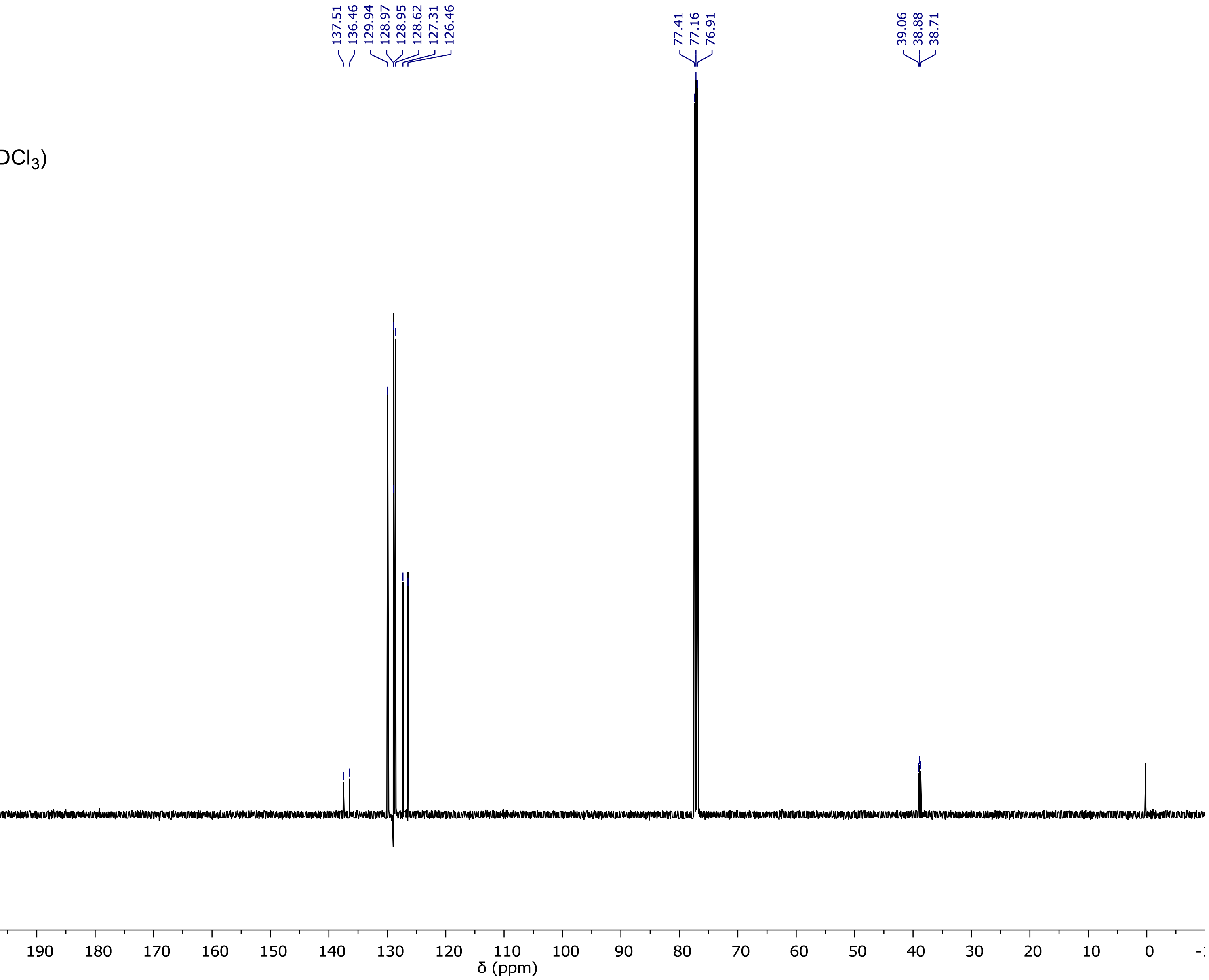

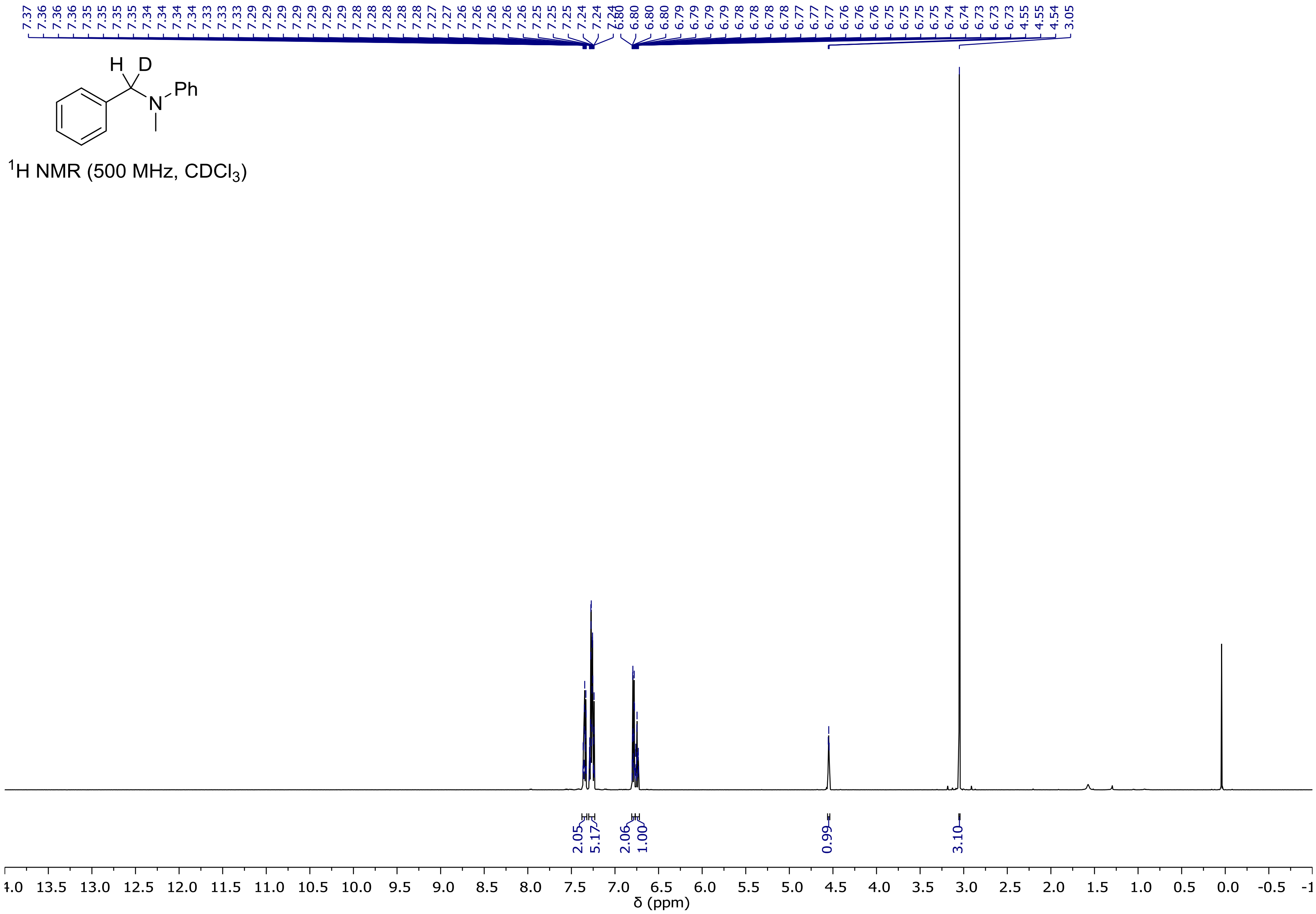
H D
N
Ph
¹H NMR (500 MHz, CDCl3)
2.05
5.17
2.06
1.00
0.99
3.10
δ (ppm)

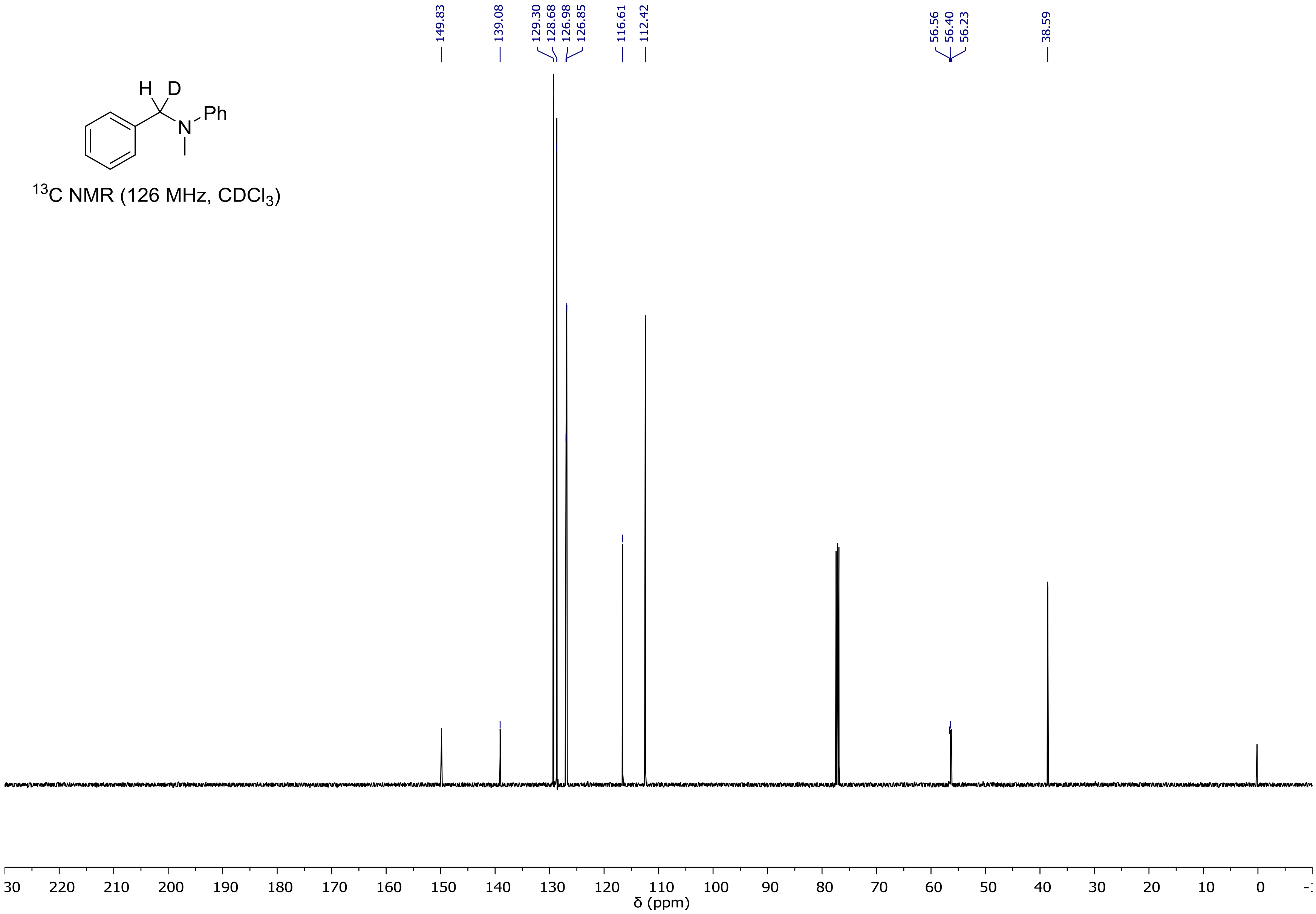

^{13}C NMR (126 MHz, $CDCl_3$)

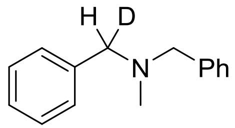

^{1}H NMR (500 MHz, $CDCl_3$)

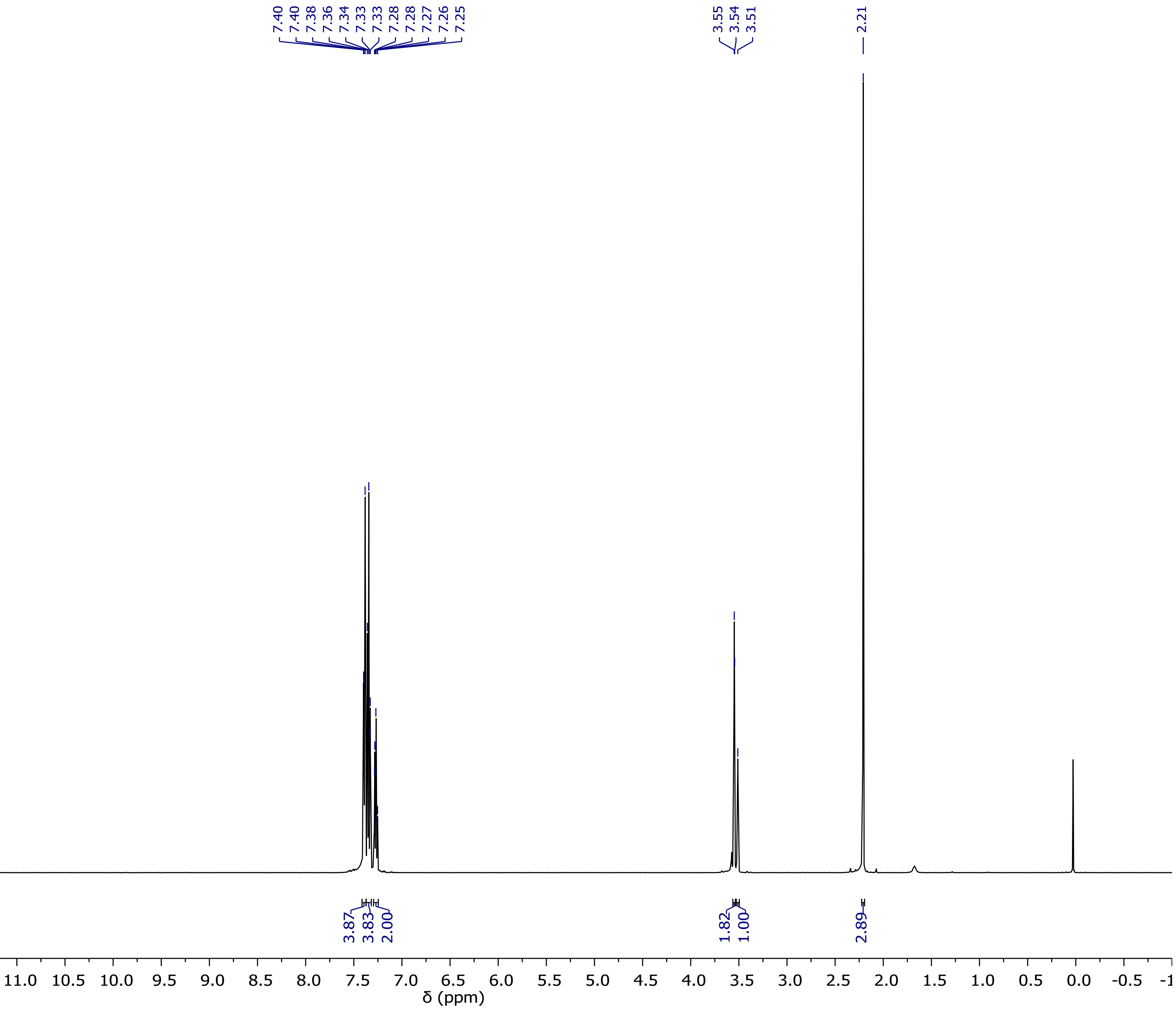

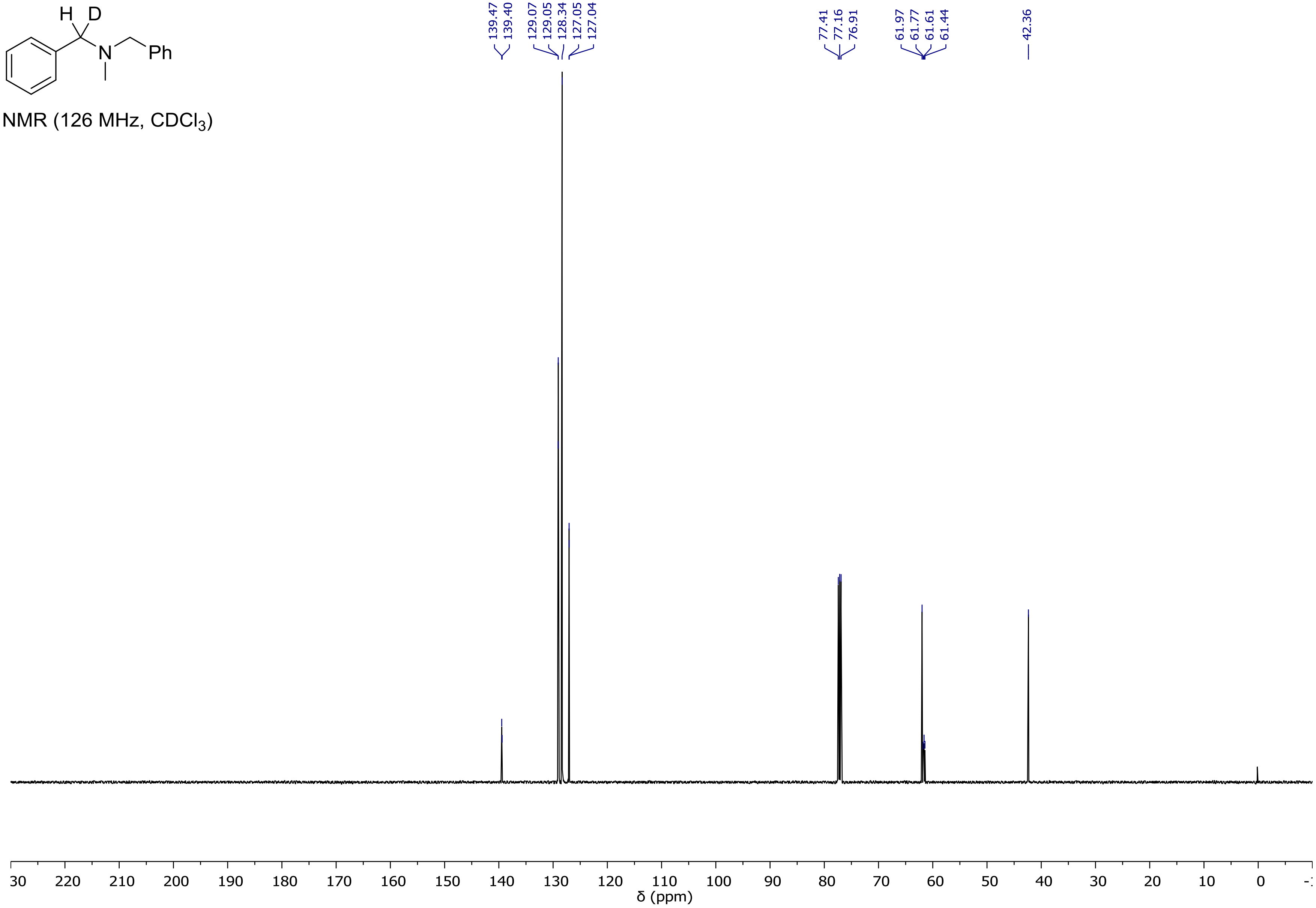

^{13}C NMR (126 MHz, $CDCl_3$)

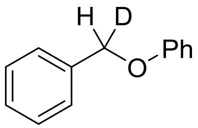

^{1}H NMR (500 MHz, $CDCl_3$)

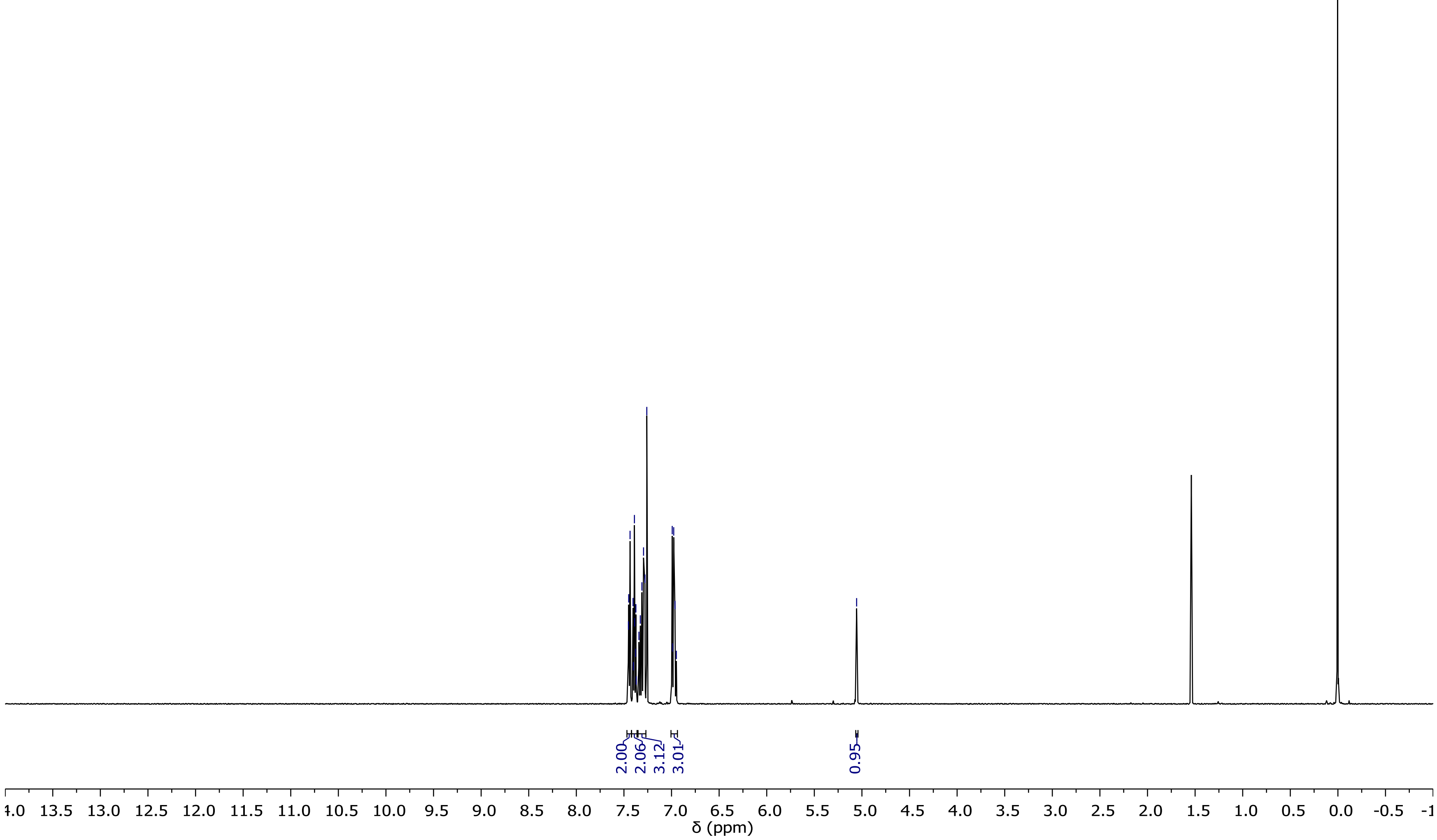

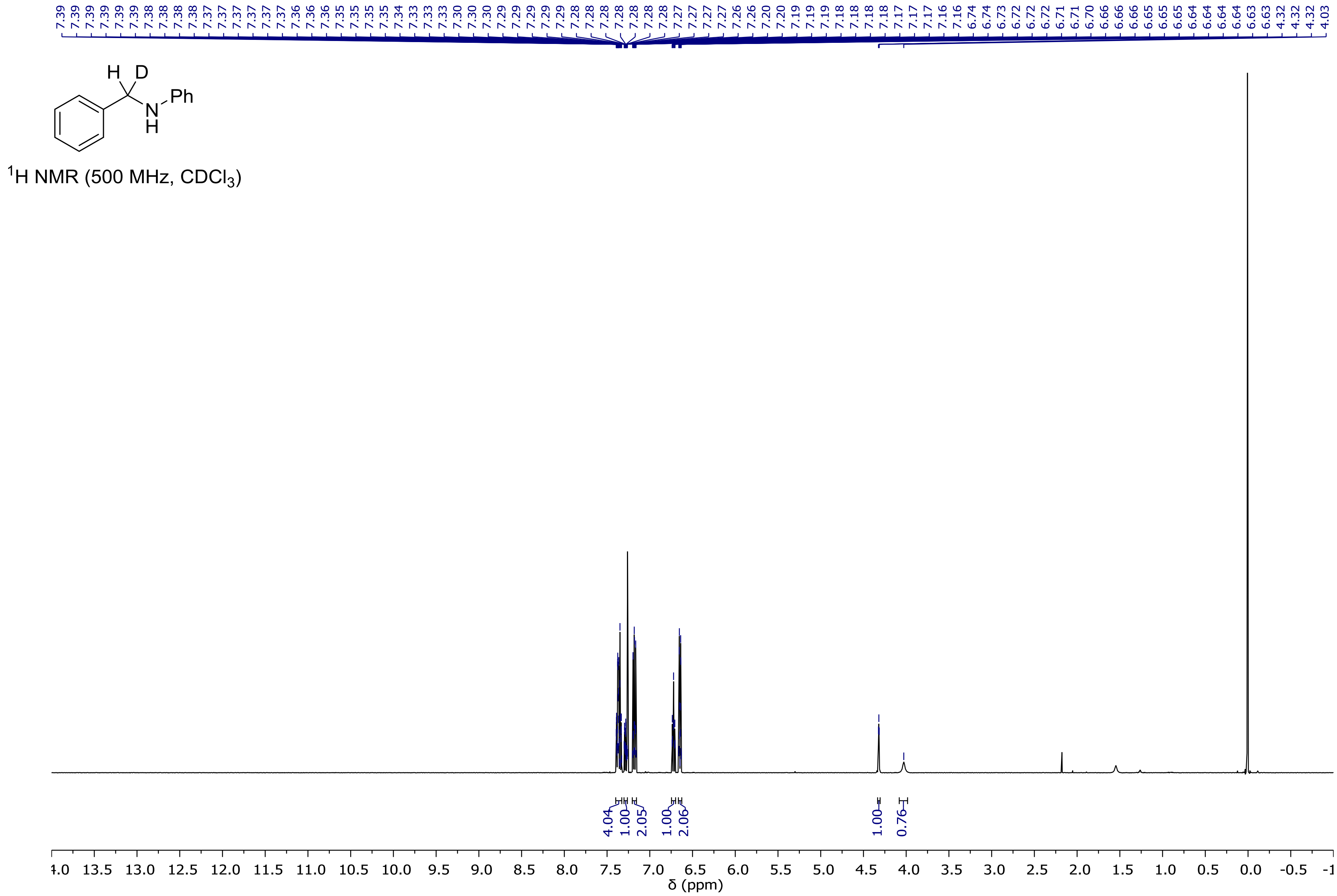
H D
N Ph
H
¹H NMR (500 MHz, CDCl₃)
δ (ppm)

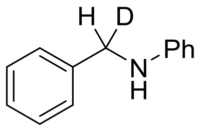

^{13}C NMR (126 MHz, $CDCl_3$)

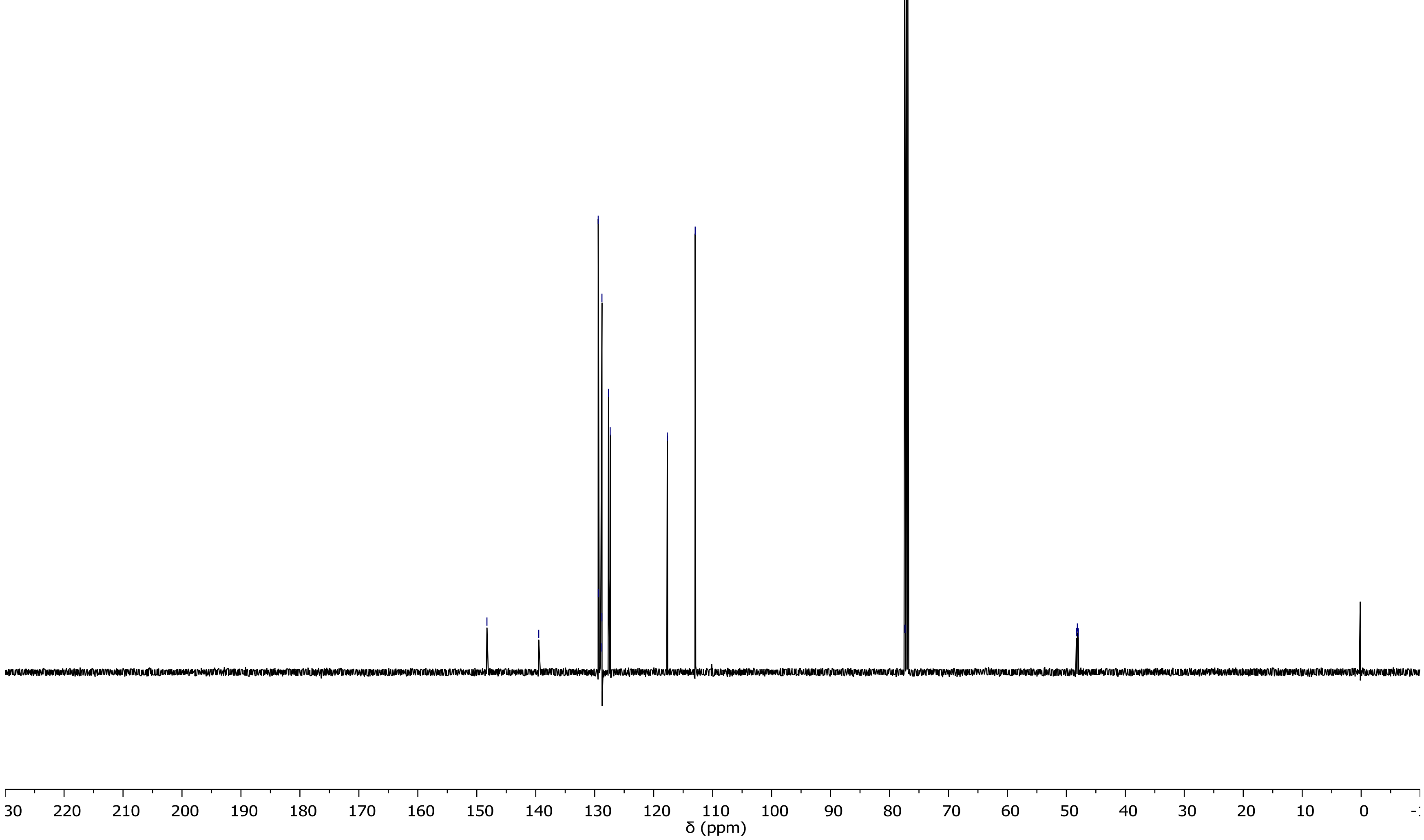

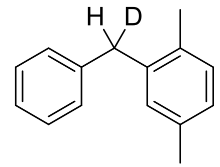

^{1}H NMR (500 MHz, $CDCl_3$)

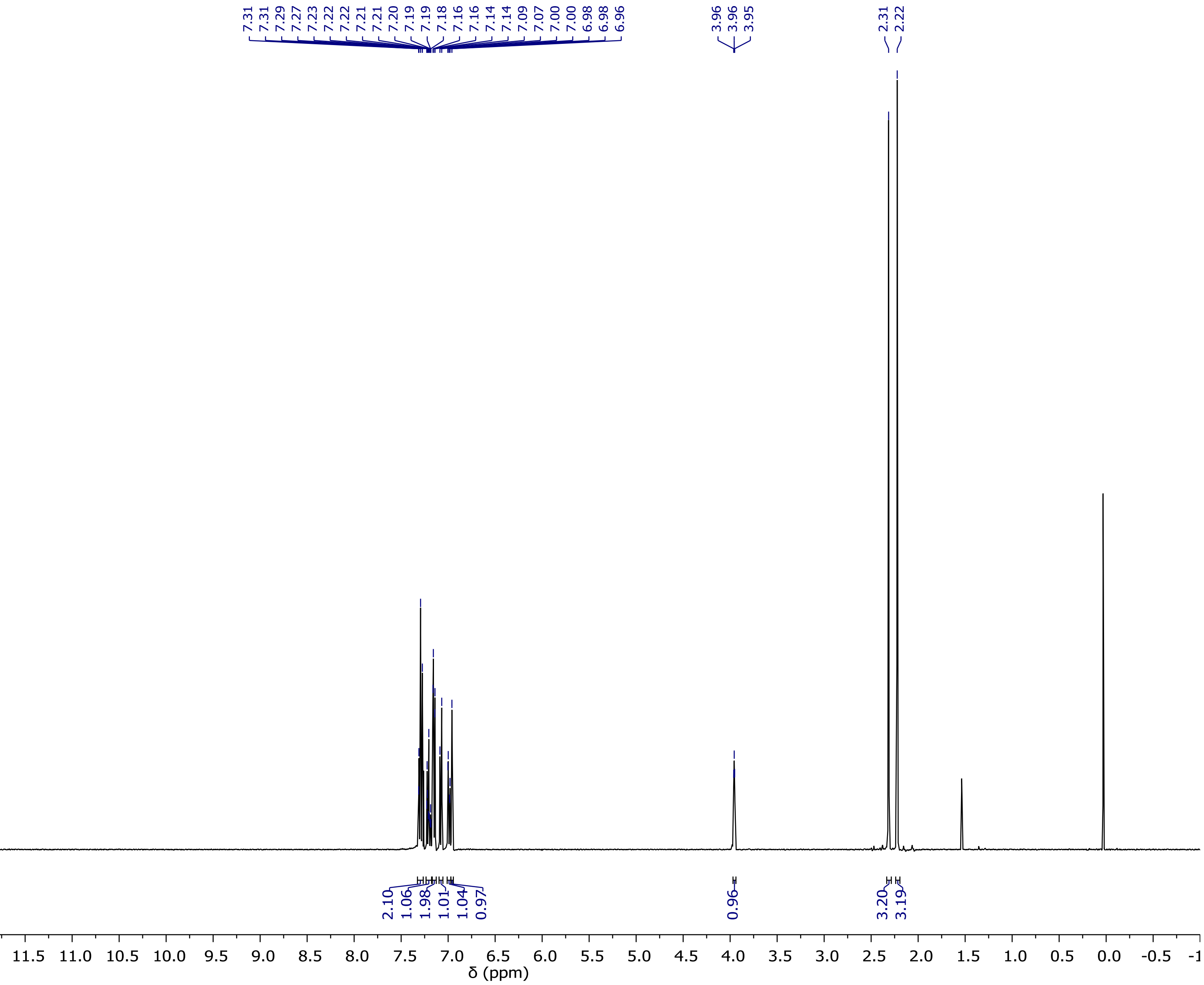

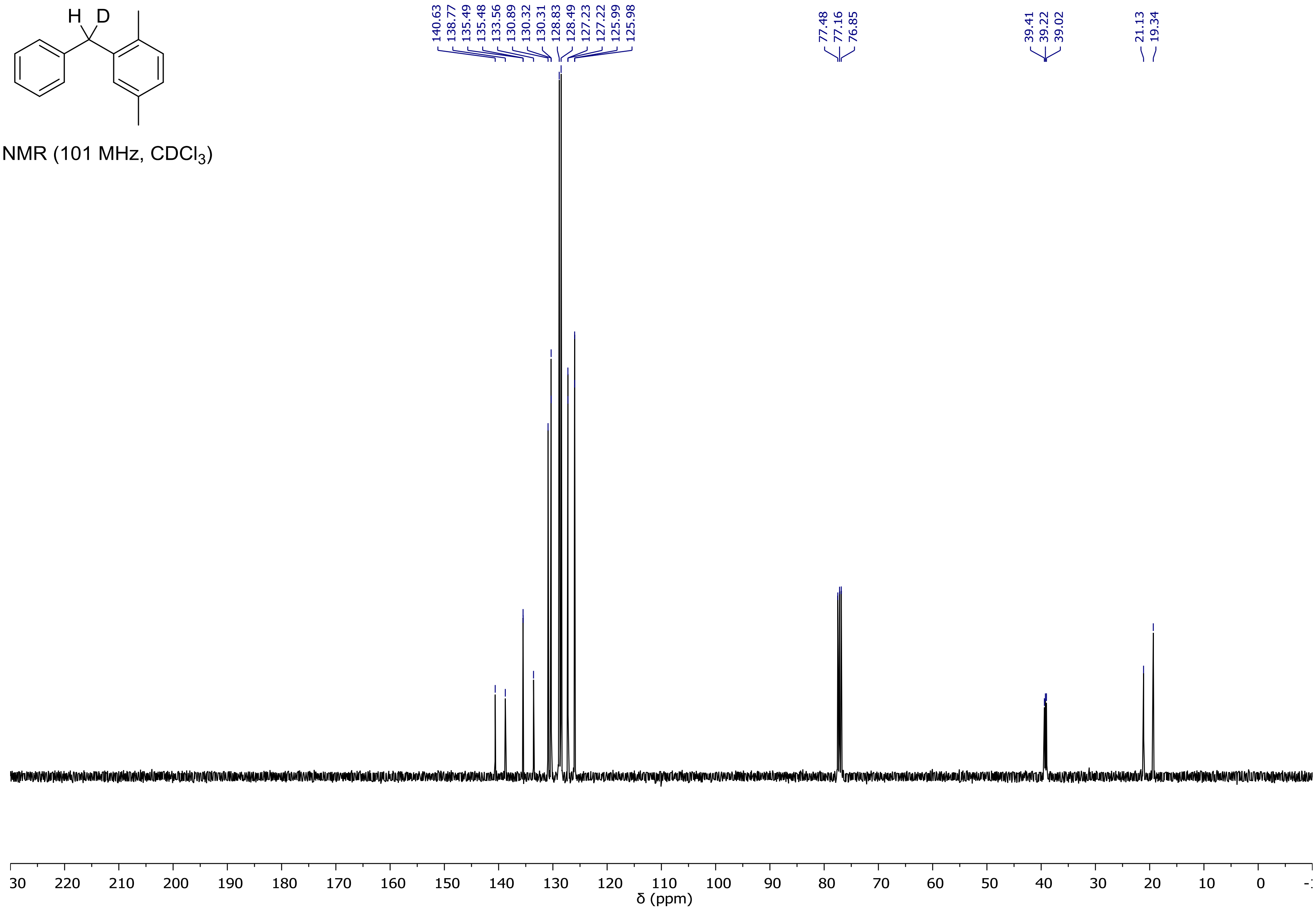

^{13}C NMR (101 MHz, $CDCl_3$)

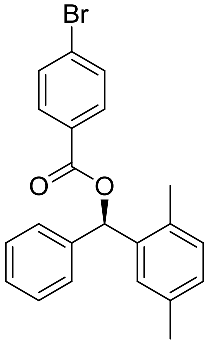

^{1}H NMR (400 MHz, $CDCl_3$)

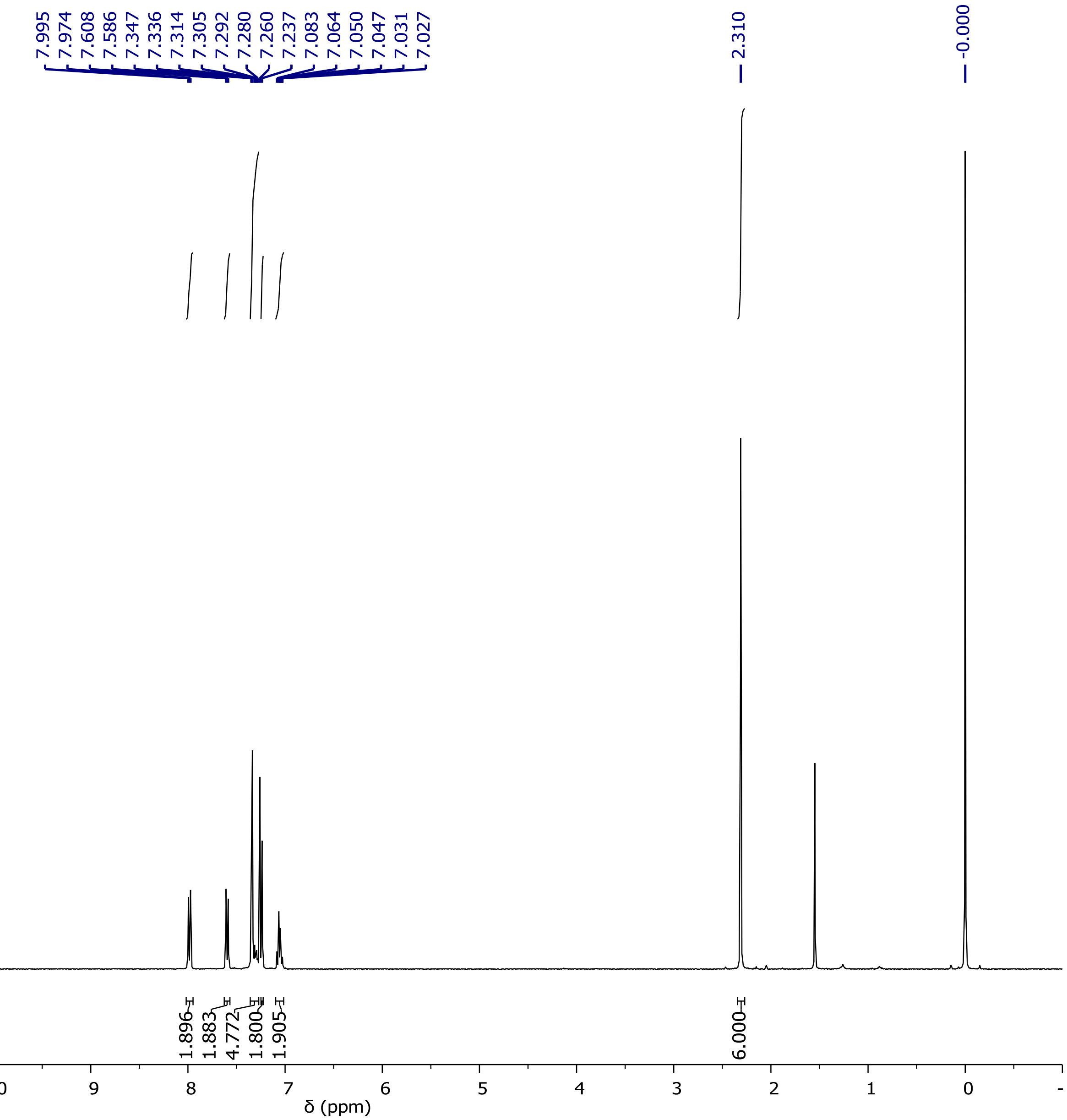

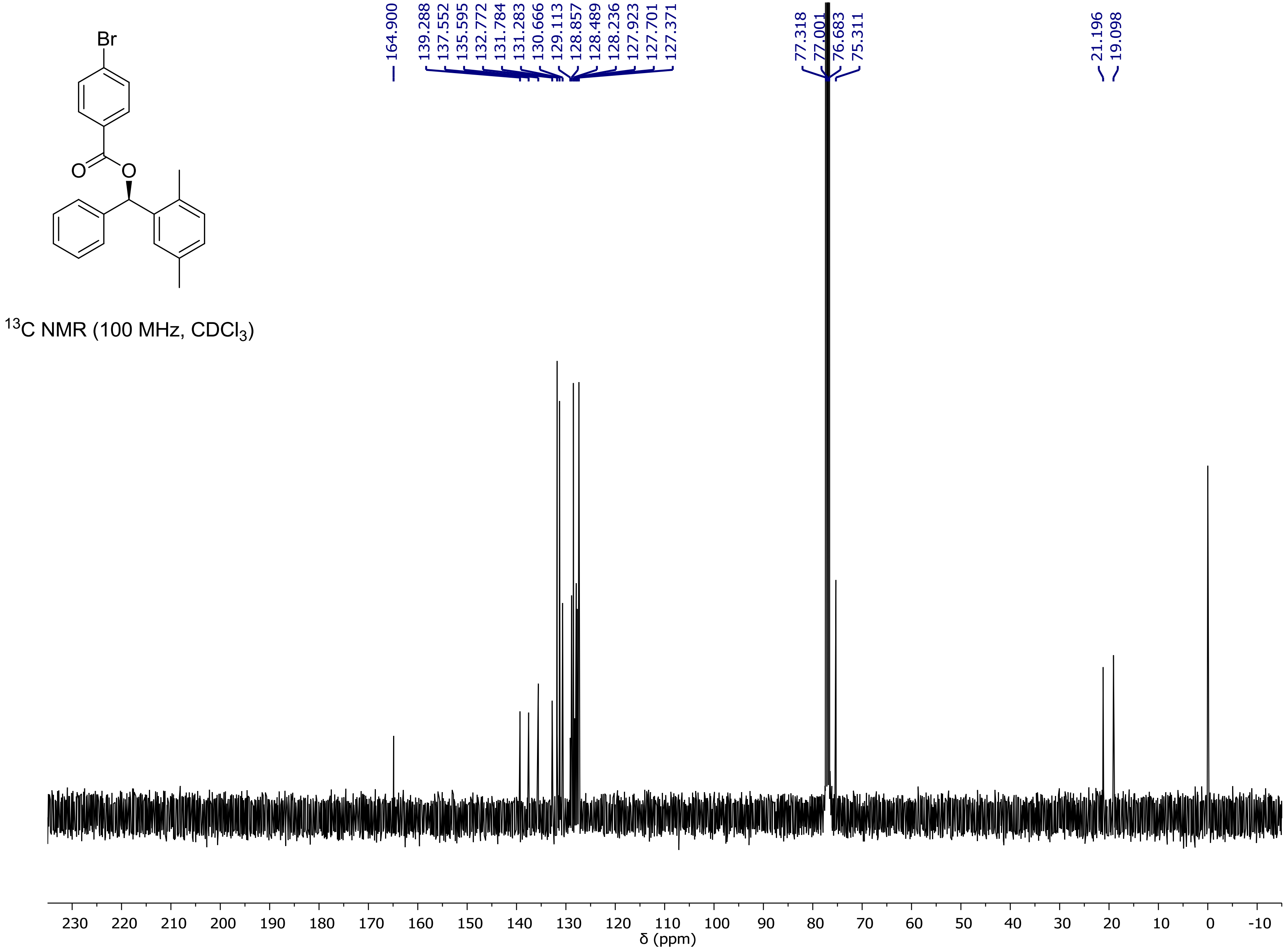

^{13}C NMR (100 MHz, $CDCl_3$)
