# Supplementary material for: Stereochemical outcomes of C–F activation reactions of benzyl fluoride
Source: Beilstein J Org Chem. 2018 Jan 9;14:106–13. doi: 10.3762/bjoc.14.6 (PMC5789430; doi:10.3762/bjoc.14.6)
Supplement: File 2 — 2H NMR analysis of enantiopurity. [file Beilstein_J_Org_Chem-14-106-s002.pdf]

Supporting Information File 2

for

Stereochemical outcomes of C–F activation reactions of benzyl fluoride

Neil S. Keddie[†,1], Pier Alexandre Champagne[†,2], Justine Desroches[2], Jean-François Paquin[*,2] and David O'Hagan[*,1]

Address: [1]School of Chemistry, Biomedical Sciences Research Complex, University of St Andrews, North Haugh, St Andrews, Fife KY16 9ST, United Kingdom and [2]PROTEO, CCVC, Département de chimie, 1045 Avenue de la Médecine, Université Laval, Québec, QC G1V 0A6, Canada

Email: Jean-François Paquin - jean-francois.paquin@chm.ulaval.ca; David O'Hagan - do1@st-andrews.ac.uk

[†] These authors contributed equally to this work.

* Corresponding author

^{2}H NMR analysis of enantiopurity

Analysis protocol

$^{2}H\{^{1}H\}$ NMR spectra were recorded at 107.5 MHz, on a Bruker Avance III-HD 700 Spectrometer, equipped with a Prodigy TCI Cryoprobe.

Poly-γ-benzyl-L-glutamate (MWT 150–350K) and spectrophotometric grade $CHCl_3$ were purchased from SigmaAldrich UK and used without further purification.

The poly-γ-benzyl-L-glutamate (γ-PBG) solutions (13% *w/w*) for analysis were prepared by addition of γ-PBG (120 mg), the analyte (approximately 20 mg) and $CHCl_3$ (800 mg) into a pre-weighed 5 mm NMR tube. The NMR tube was sealed with a PTFE pressure cap (Wilmad 521-PC) and left to stand for 1 h. The NMR tube was placed in a plastic tube and it was spun manually with a cord to achieve a centrifugal force, drawing the gel from one end of the tube to the other. The tube was inverted and this manual centrifugation was repeated 40 times, or further if complete dissolution of the polymer was not achieved. The samples were left to stand for a further 1 h to equilibrate, prior to acquiring the $^{2}H\{^{1}H\}$ NMR spectrum.

Enantiomeric excess (ee) values were determined by deconvolution of the line shapes of the $^{2}H\{^{1}H\}$ NMR spectrum in mNova 11.0.4 and subsequent integration. The mean value of the two signals relating to each enantiomer was used to calculate the ee.

Scheme 1 (2 → 3)

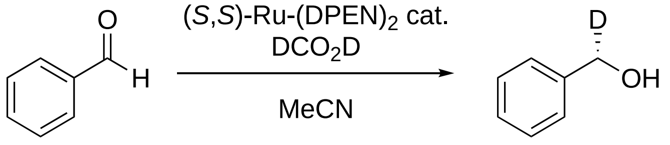 **2** 81% **(*S*)-3**	**ee** 95%

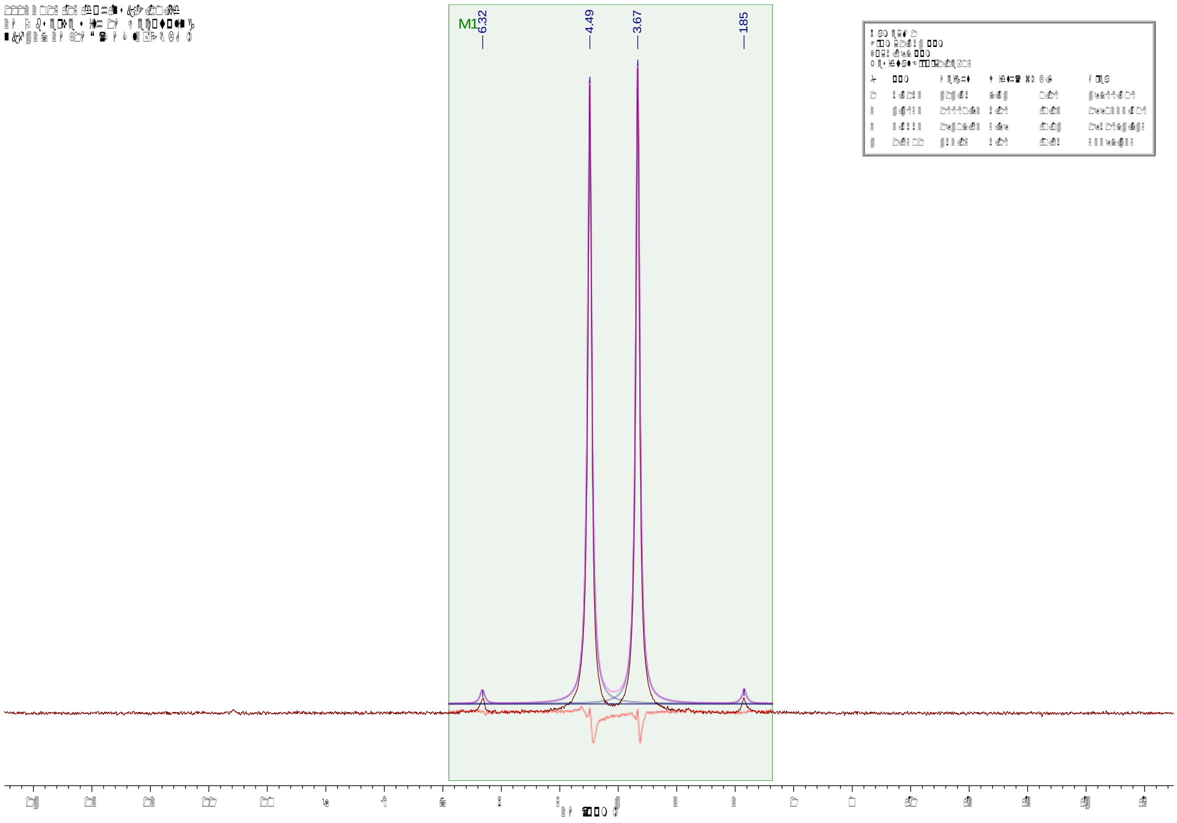

$^2H\{^1H\}$ NMR (76.7 MHz) with PBLG in $CHCl_3$ (13% *w/w*) of (*S*)-**3**.

Scheme 1 (3 → 1)

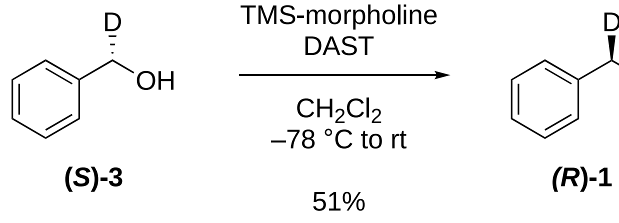

ee

94%

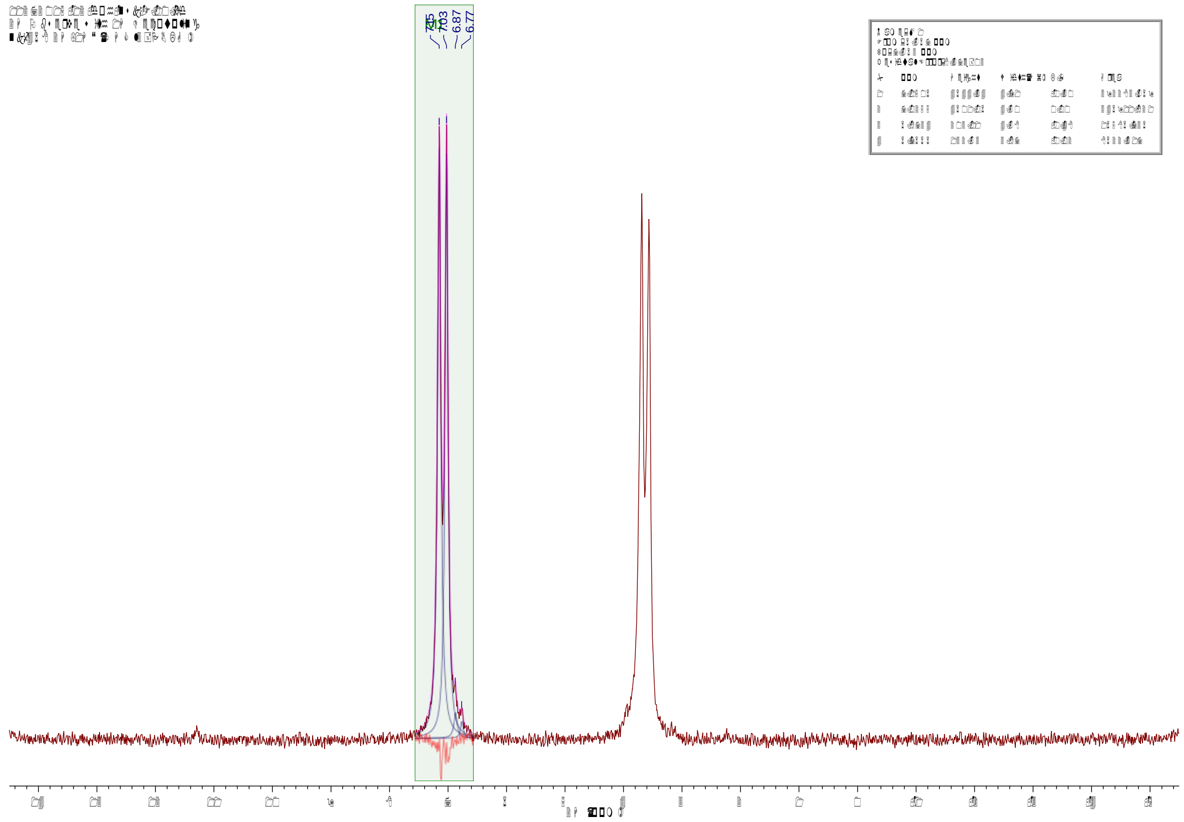

^{2}H{^{1}H} NMR (76.7 MHz) with PBLG in $CHCl_3$ (13% *w/w*) of (*R*)-**1**.

Table 1, entry 1

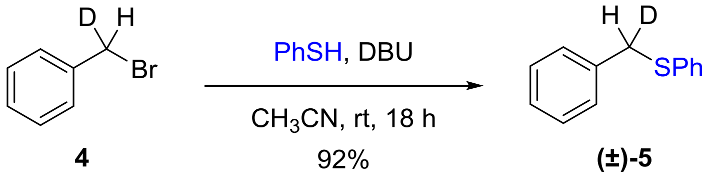

	**ee**
	racemic

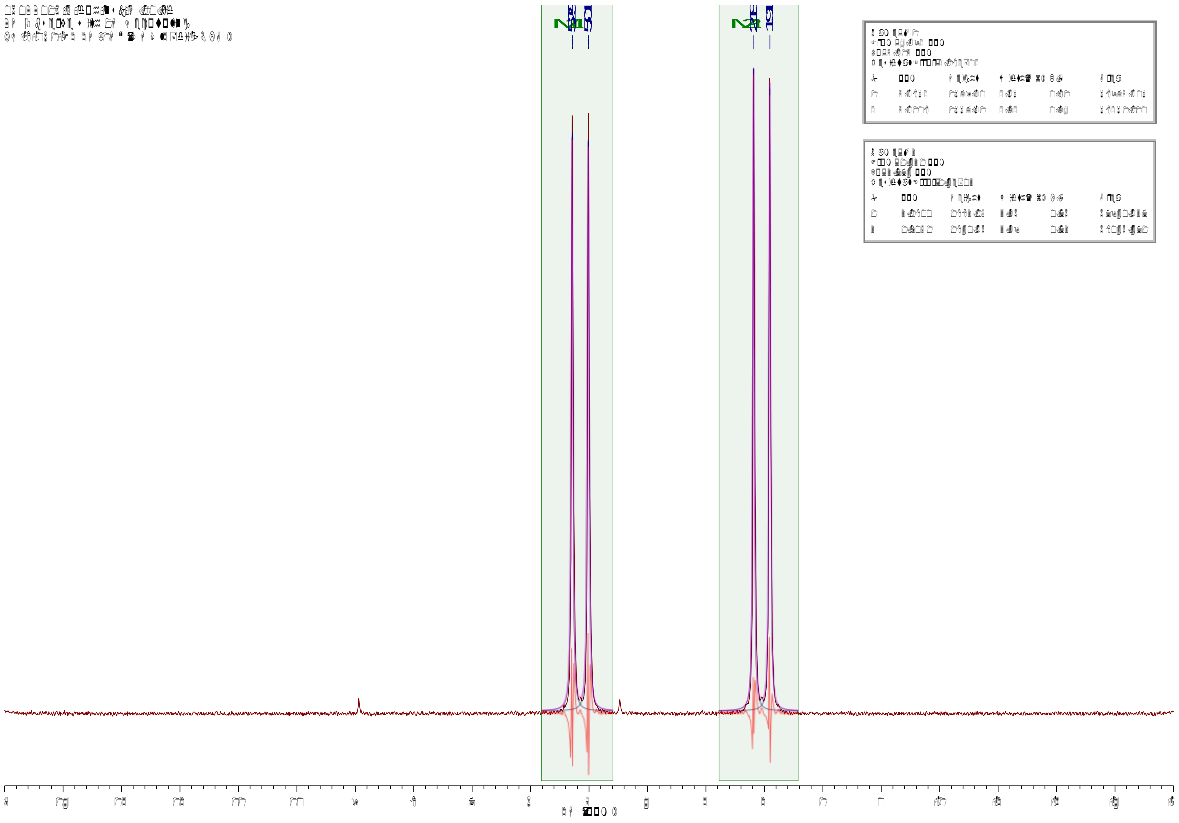

$^{2}H\{^{1}H\}$ NMR (107.5 MHz) with PBLG in $CHCl_3$ (13% *w/w*) of (±)-**5**.

Table 1, entry 2

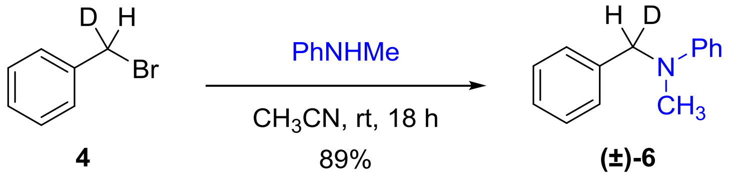	**ee**<br>racemic

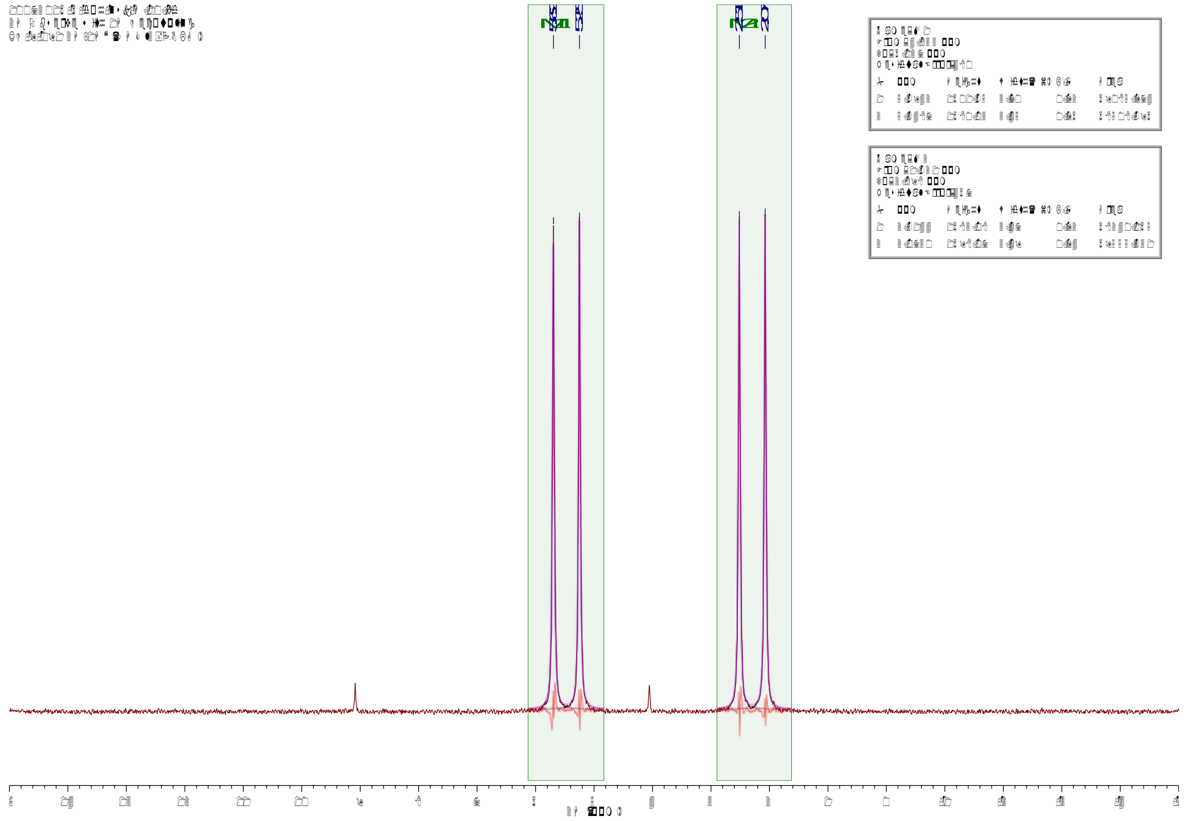

$^2H\{^1H\}$ NMR (107.5 MHz) with PBLG in $CHCl_3$ (13% *w/w*) of (±)-**6**.

Table 1, entry 3

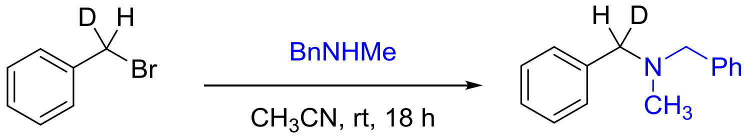 **4** 69% **(±)-7**	nd[a]

[a] ee could not be determined as a result of poor $^{2}H\{^{1}H\}$ NMR resolution

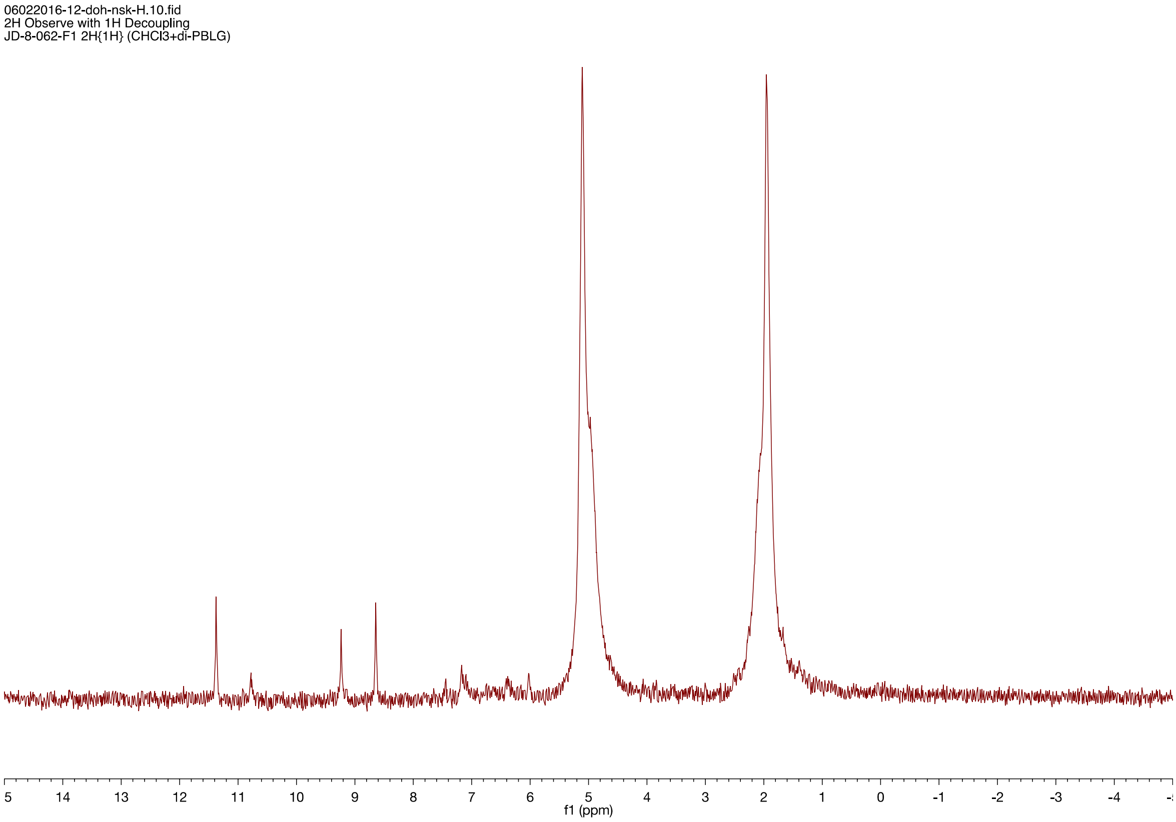

$^{2}H\{^{1}H\}$ NMR (107.5 MHz) with PBLG in $CHCl_3$ (13% *w/w*) of (±)-**7**.

Table 2, entry 1

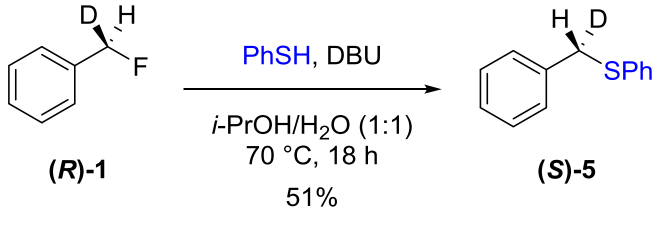	**ee**<br>94%

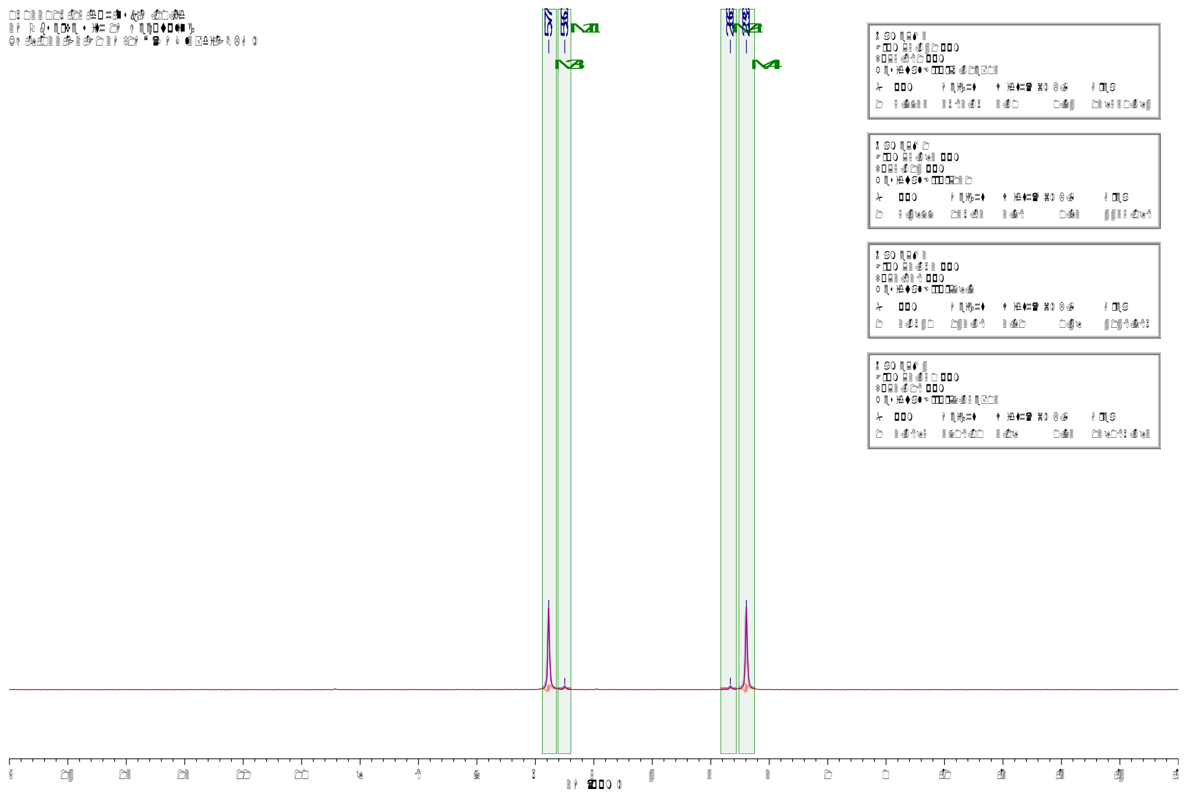

^{2}H{^{1}H} NMR (107.5 MHz) with PBLG in $CHCl_3$ (13% *w/w*) of (*S*)-**5**.

Table 2, entry 2

	ee
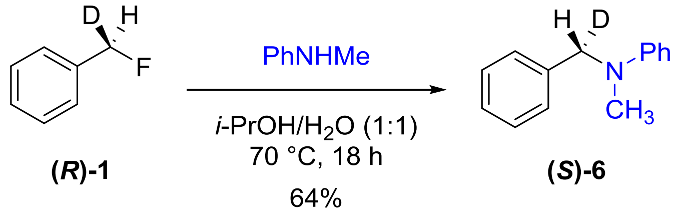	90%

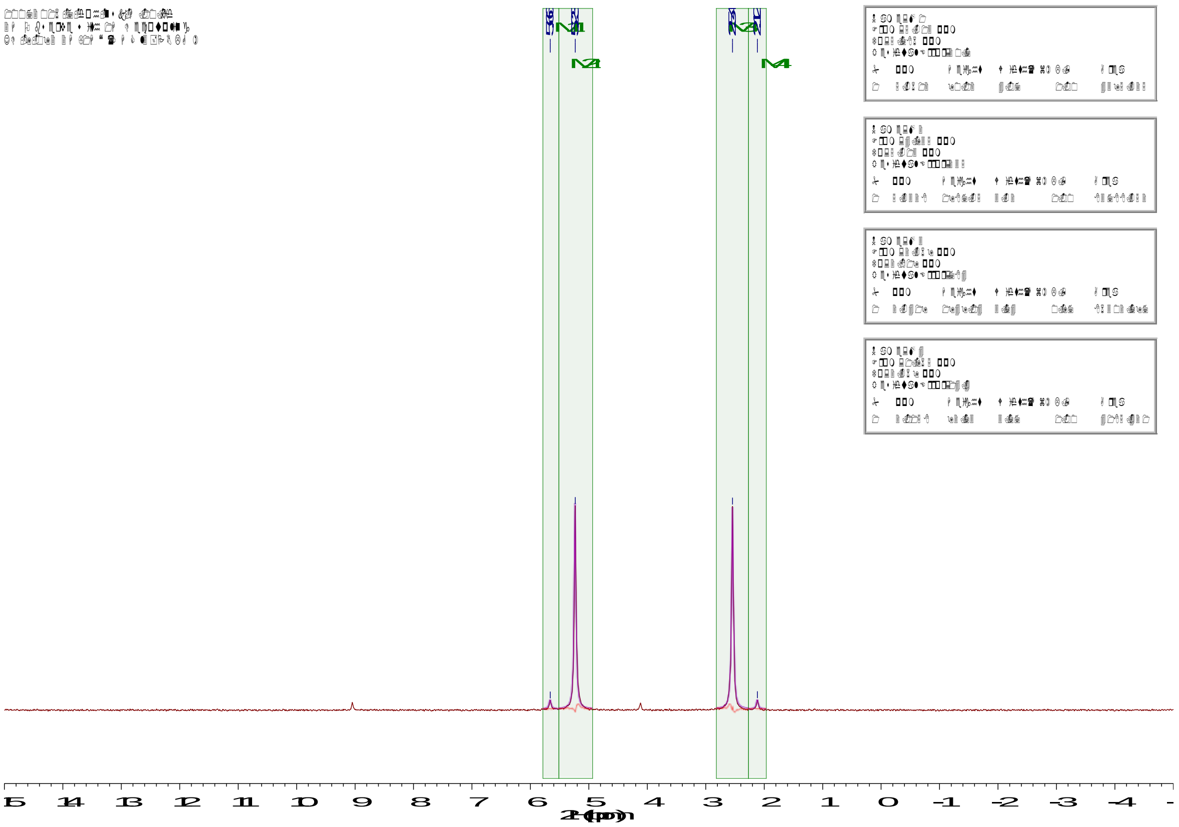

$^{2}H\{^{1}H\}$ NMR (107.5 MHz) with PBLG in $CHCl_3$ (13% *w/w*) of (*S*)-**6**.

Table 2, entry 3

	ee
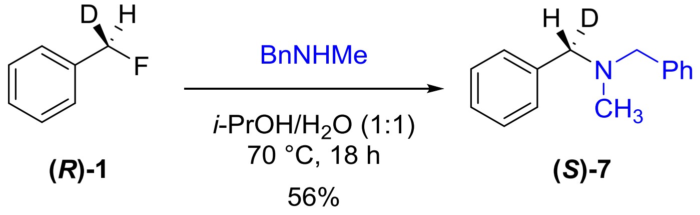	nd[a]

[a] ee could not be determined as a result of poor $^{2}H\{^{1}H\}$ NMR resolution

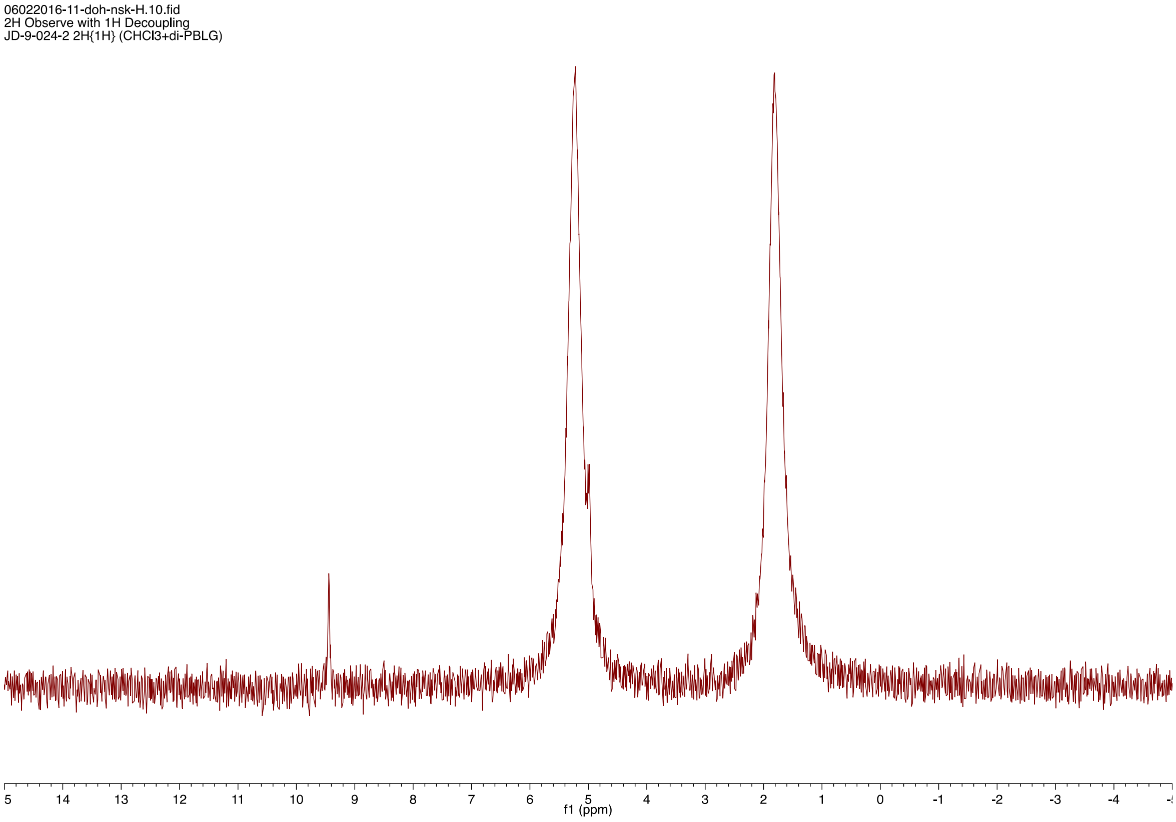

$^{2}H\{^{1}H\}$ NMR (107.5 MHz) with PBLG in $CHCl_3$ (13% *w/w*) of (*S*)-**7**.

Table 2, entry 4

	ee
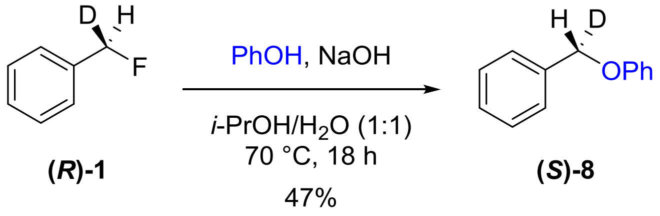	93%

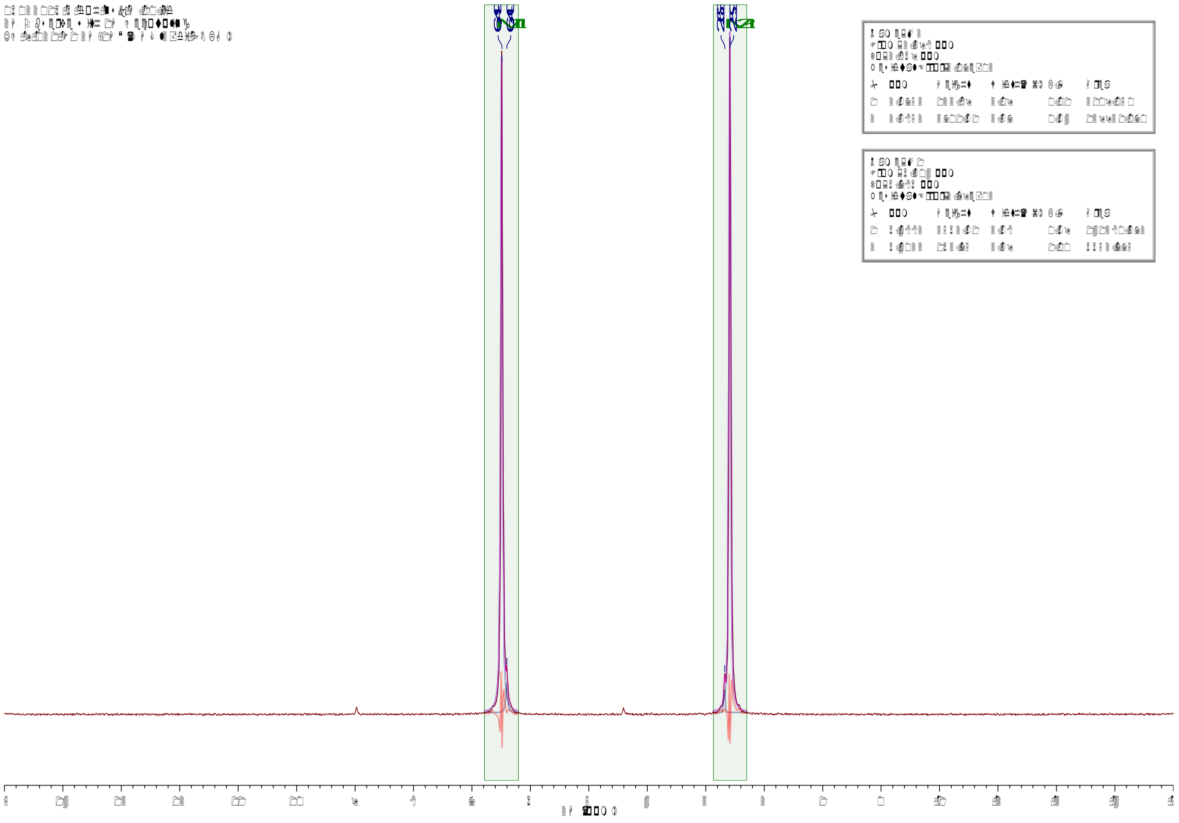

$^2H\{^1H\}$ NMR (107.5 MHz) with PBLG in $CHCl_3$ (13% *w/w*) of (*S*)-**8**.

Table 2, entry 5

	ee
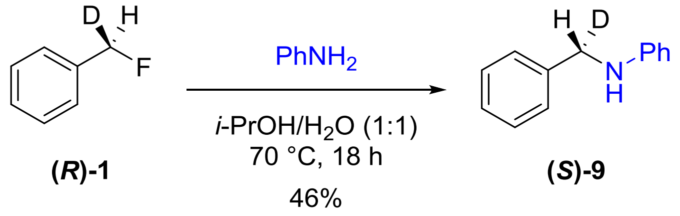	91%

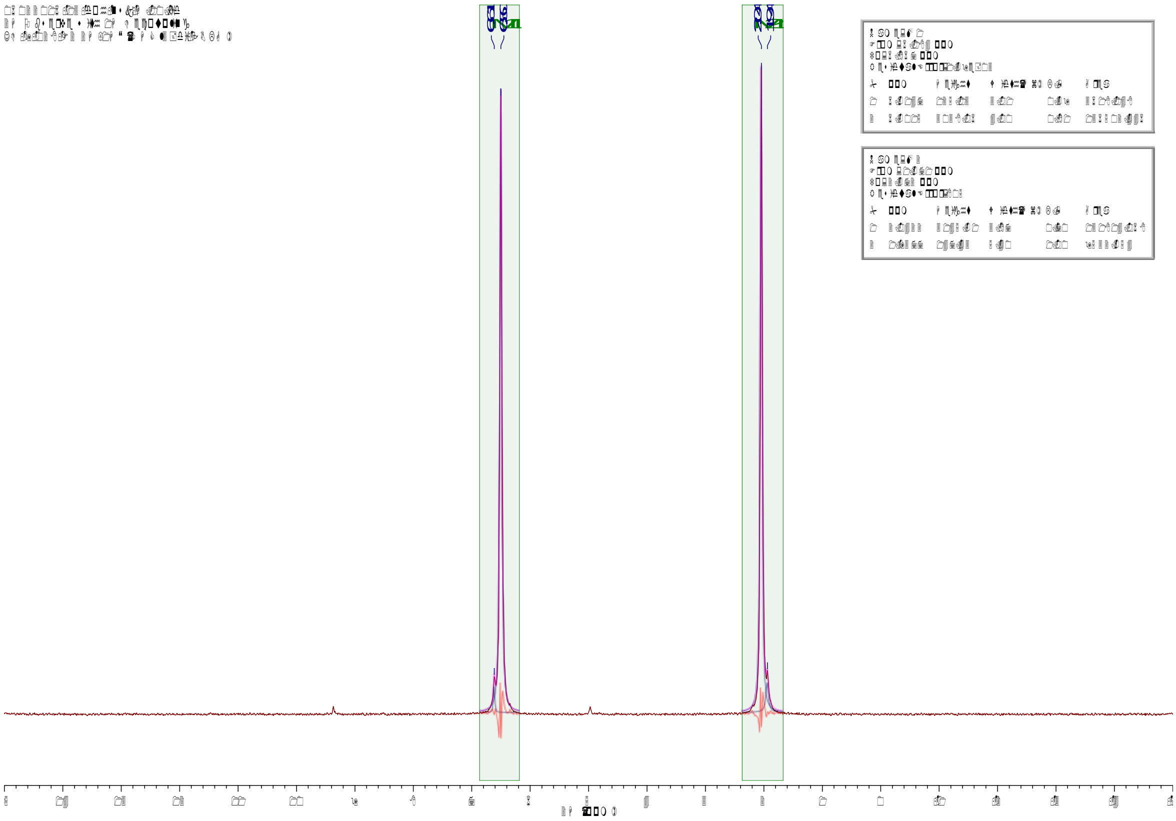

^{2}H{^{1}H} NMR (107.5 MHz) with PBLG in $CHCl_3$ (13% *w/w*) of (*S*)-**9**.

Table 2, entry 6

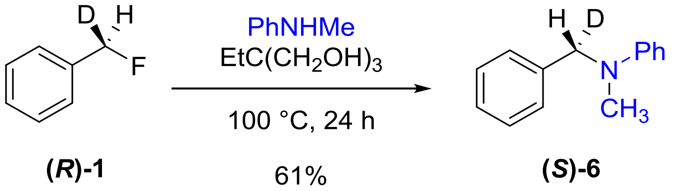

ee
87%

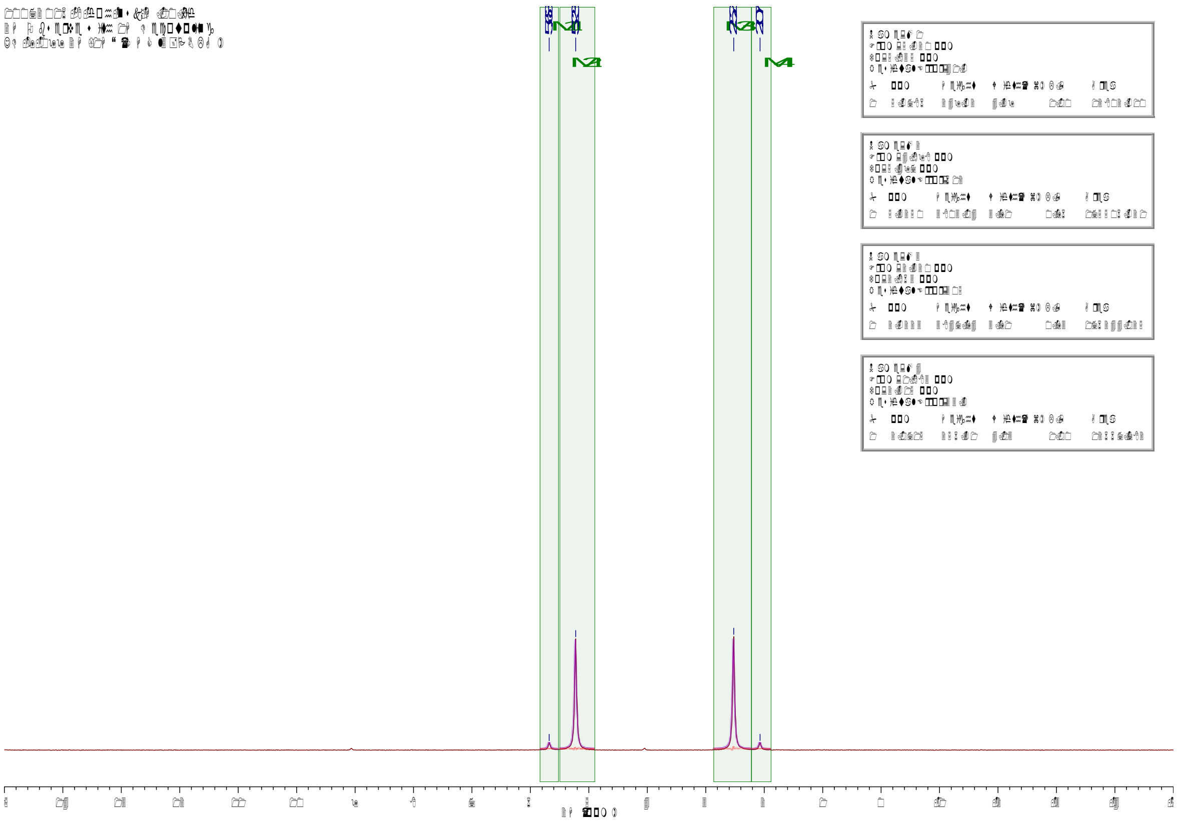

$^2H\{^1H\}$ NMR (107.5 MHz) with PBLG in $CHCl_3$ (13% *w/w*) of (*S*)-**6**.

Table 2, entry 7

	ee
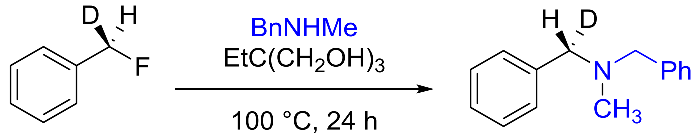 (*R*)-**1** 70% (*S*)-**7**	nd[a]

[a] ee could not be determined as a result of poor $^{2}H\{^{1}H\}$ NMR resolution

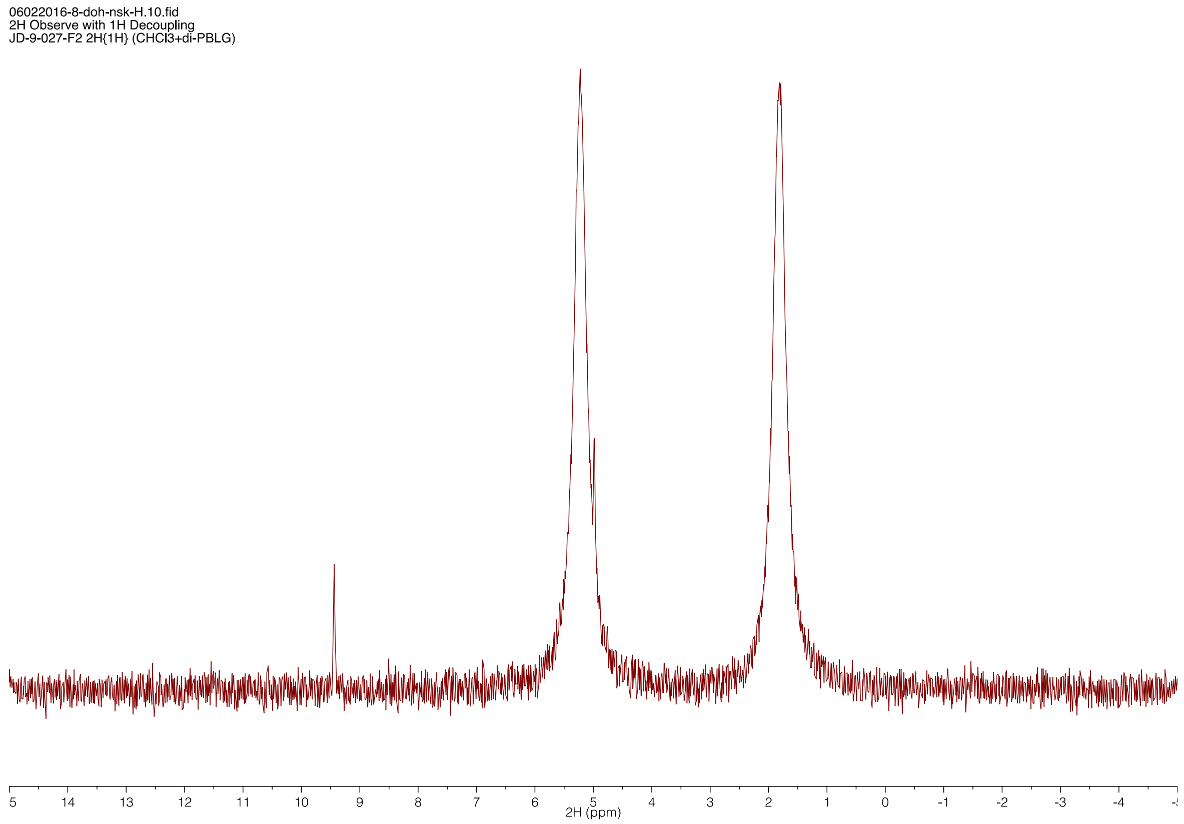

$^{2}H\{^{1}H\}$ NMR (107.5 MHz) with PBLG in $CHCl_3$ (13% *w/w*) of (*S*)-**7**.

Table 2, entry 8

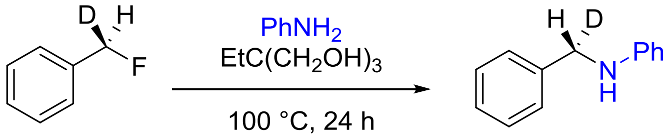

**(*R*)-1** 25% **(*S*)-9**	**ee** 89%

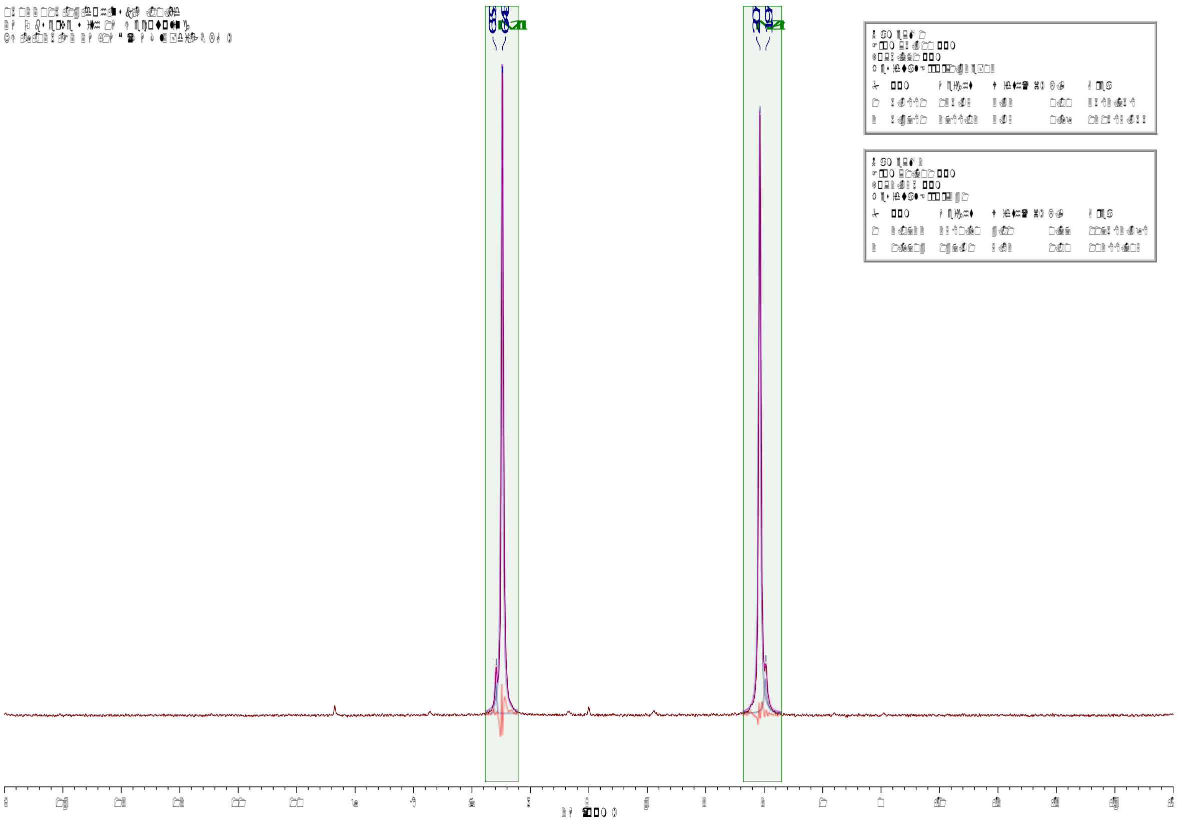

$^2H\{^1H\}$ NMR (107.5 MHz) with PBLG in $CHCl_3$ (13% *w/w*) of (*S*)-**9**.

Table 3, entry 1

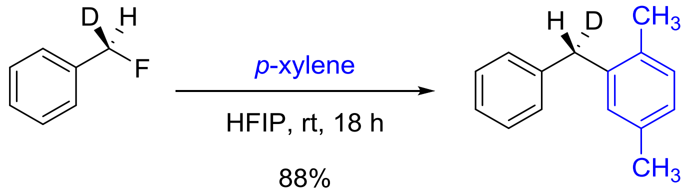

(***R***)**-1** (***S***)**-10**

ee

24%

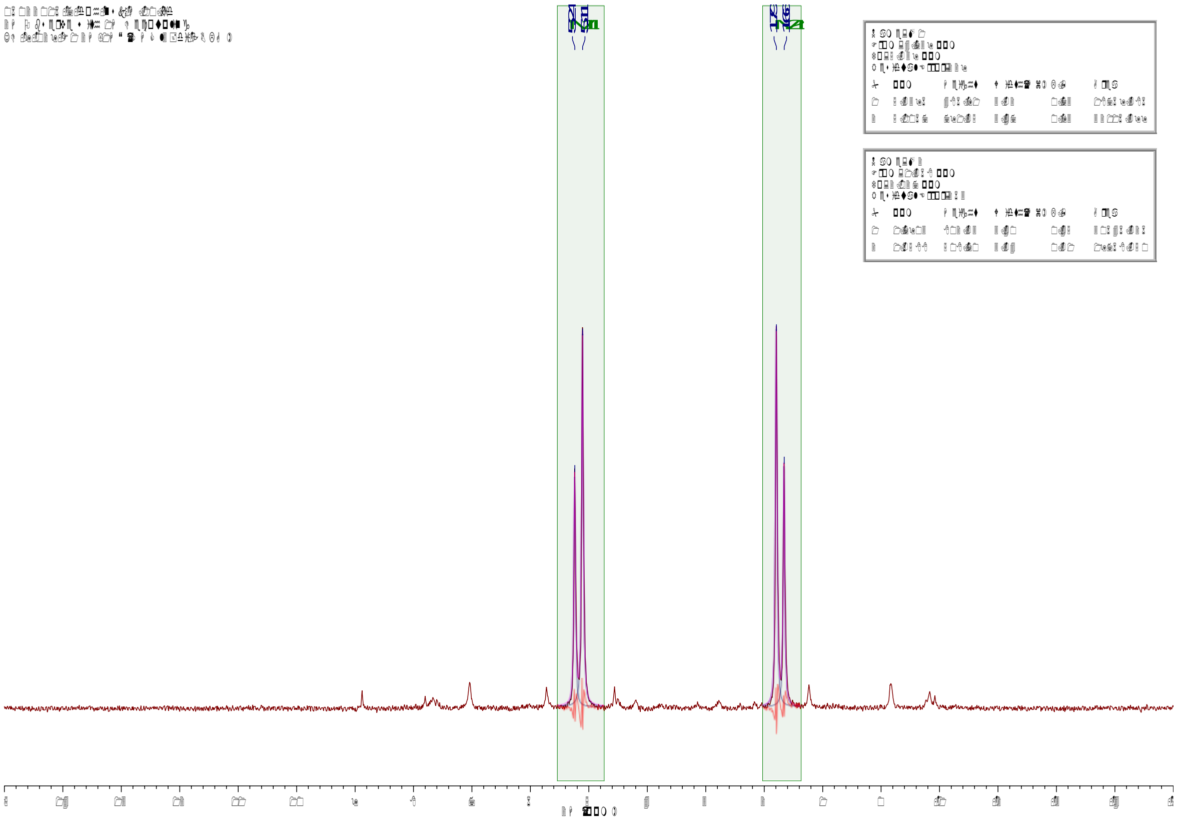

$^2H\{^1H\}$ NMR (107.5 MHz) with PBLG in $CHCl_3$ (13% *w/w*) of (*S*)-**10**.

Table 3, entry 2

	ee
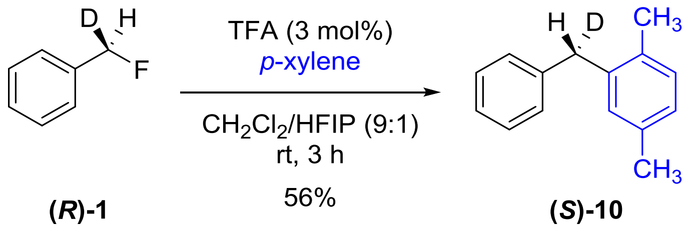	19%

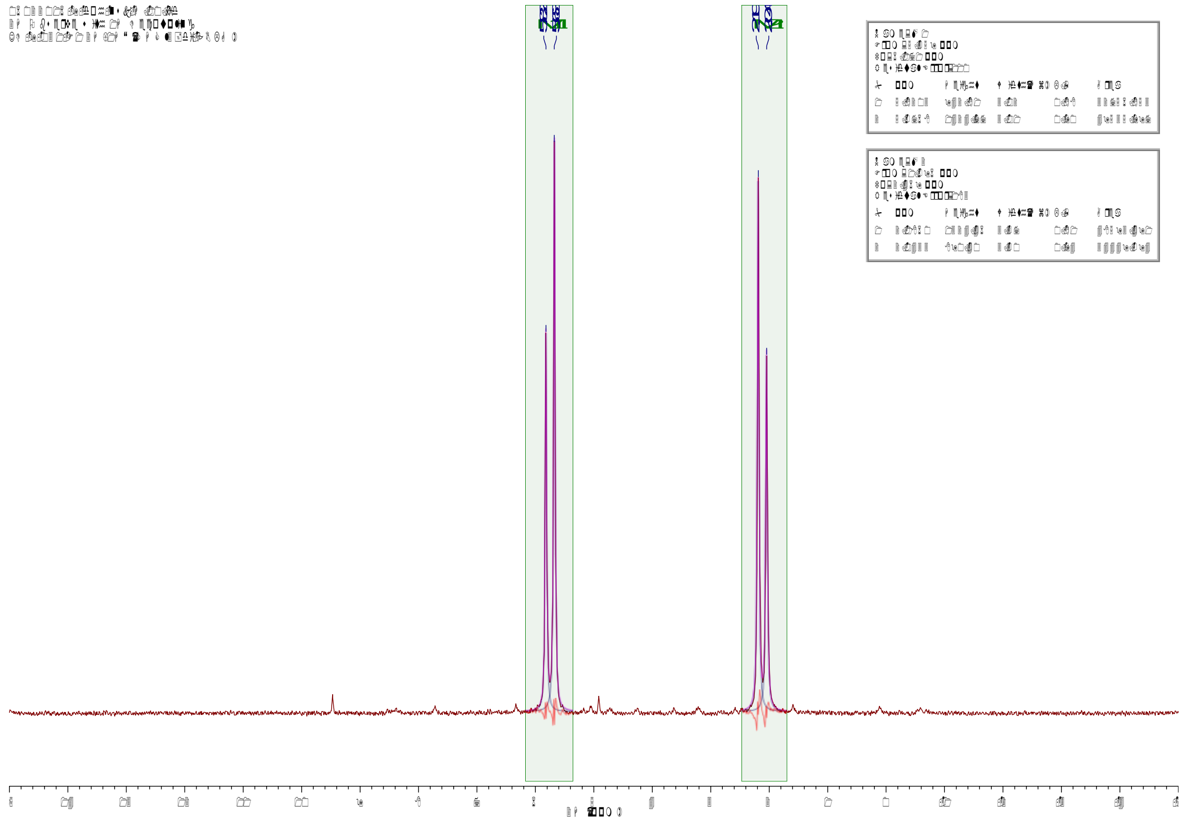

^{2}H{^{1}H} NMR (107.5 MHz) with PBLG in $CHCl_3$ (13% *w/w*) of (*S*)-**10**.

Table 3, entry 3

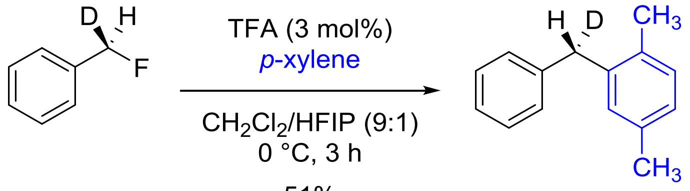 (***R***)-**1** → (***S***)-**10**	**ee** 28%

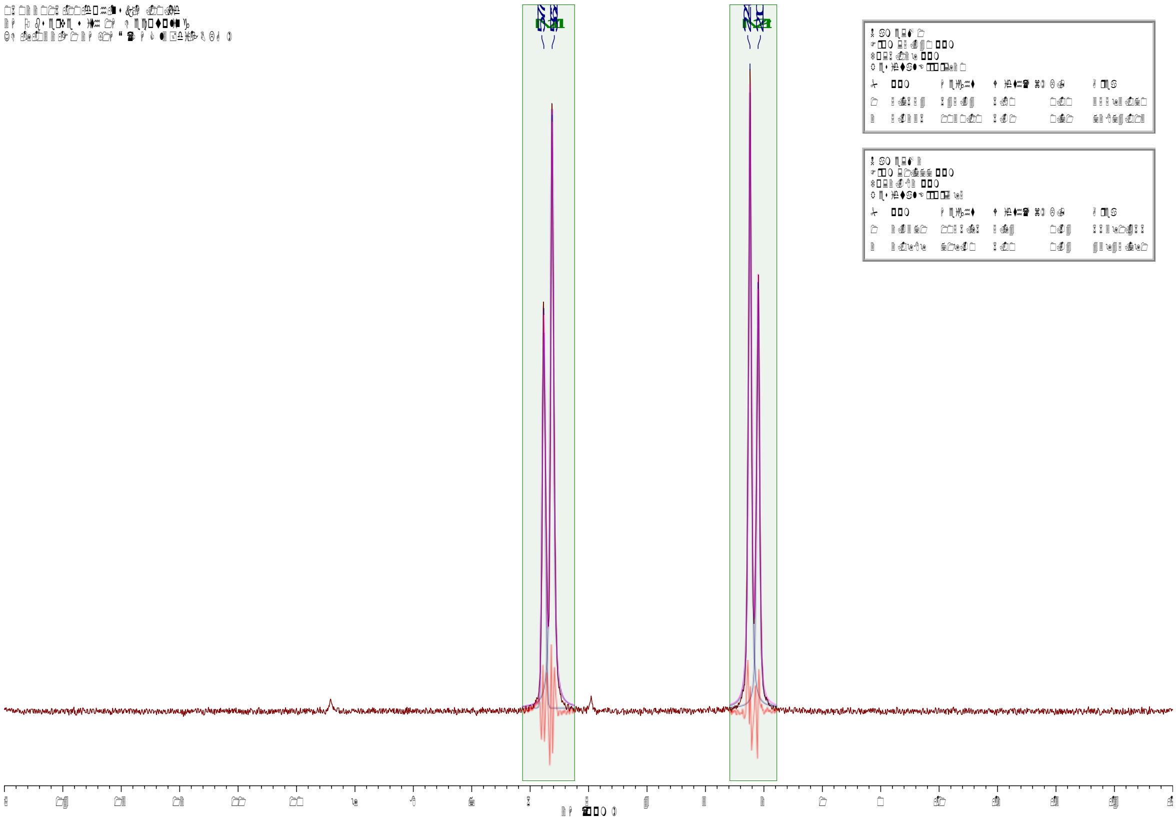

$^{2}H\{^{1}H\}$ NMR (107.5 MHz) with PBLG in $CHCl_3$ (13% *w/w*) of (*S*)-**10**.

Scheme 2 (12 → 10)

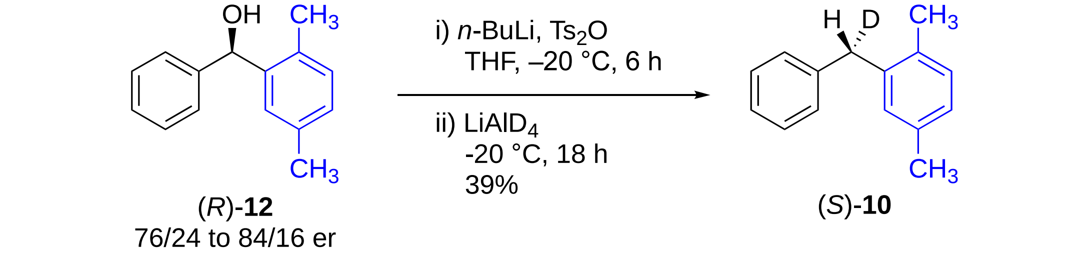

ee

18%

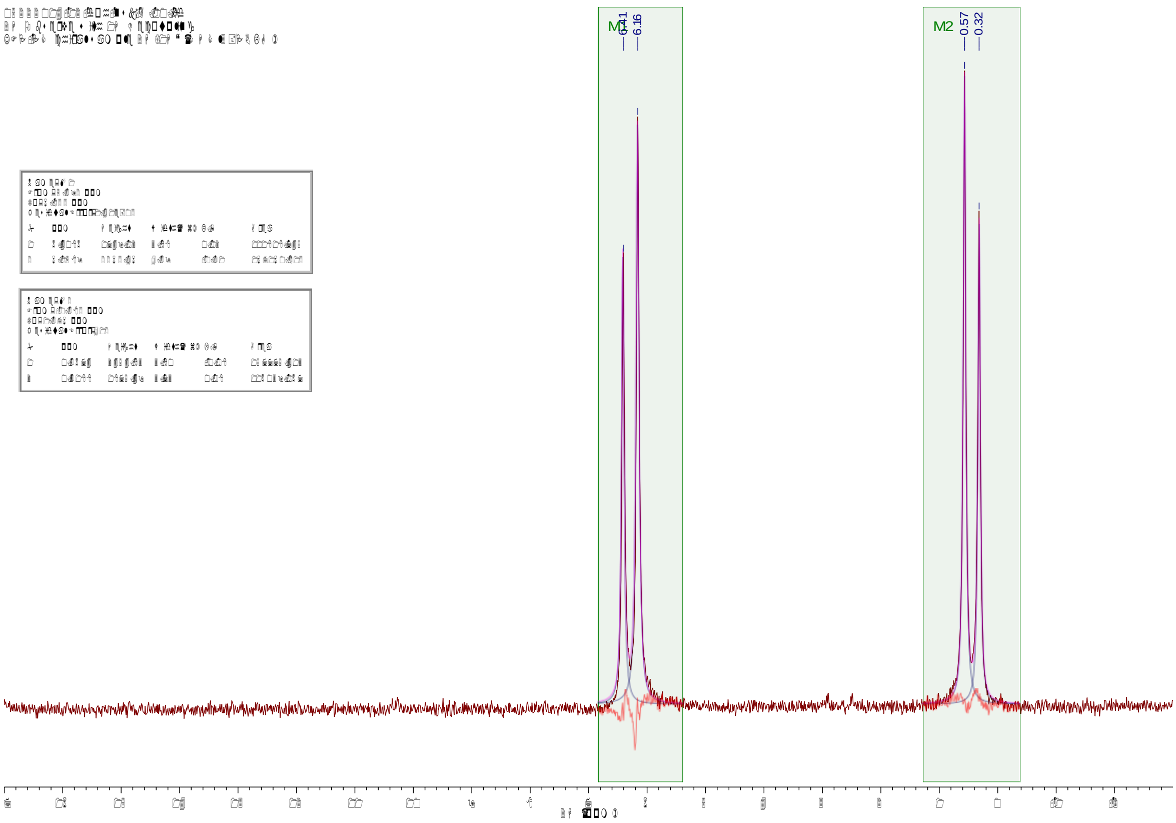

$^{2}H\{^{1}H\}$ NMR (76.7 MHz) with PBLG in $CHCl_3$ (13% *w/w*) of (*S*)-**10**.
